# A lentiviral vector expressing a dendritic cell-targeting multimer induces mucosal anti-mycobacterial CD4^+^ T-cell immunity

**DOI:** 10.1038/s41385-022-00566-z

**Published:** 2022-09-14

**Authors:** François Anna, Jodie Lopez, Fanny Moncoq, Catherine Blanc, Pierre Authié, Amandine Noirat, Ingrid Fert, Philippe Souque, Fabien Nevo, Alexandre Pawlik, David Hardy, Sophie Goyard, Denis Hudrisier, Roland Brosch, Françoise Guinet, Olivier Neyrolles, Pierre Charneau, Laleh Majlessi

**Affiliations:** 1 Pasteur-TheraVectys Joint Lab, Institut Pasteur, Université Paris Cité, 28 rue du Dr. Roux, F-75015 Paris, France; 2 Integrated Mycobacterial Pathogenomics Unit, CNRS UMR 3525, Institut Pasteur, Université Paris Cité, 25 rue du Dr. Roux, F-75015 Paris, France; 3 Histopathology Platform, Institut Pasteur, Université Paris Cité, 28 rue du Dr. Roux, F-75015 Paris, France; 4 Platform for Innovation and Development of Diagnostic Tests, Institut Pasteur, Université Paris Cité, 28 rue du Dr. Roux, F-75015 Paris, France; 5 Institut de Pharmacologie et de Biologie Structurale, IPBS, CNRS, UPS, Université de Toulouse, Toulouse, France; 6 Lymphocytes and Immunity Unit, INSERM U1223, Institut Pasteur, Université Paris Cité, 25 rue du Dr. Roux, F-75015 Paris, France

## Abstract

Most viral vectors, including the potently immunogenic lentiviral vectors (LVs), only poorly direct antigens to the MHC-II endosomal pathway and elicit CD4^+^ T cells. We developed a new generation of LVs encoding antigen-bearing monomers of collectins substituted at their C-terminal domain with the CD40 ligand ectodomain to target and activate antigen-presenting cells. Host cells transduced with such optimized LVs secreted soluble collectin-antigen polymers with the potential to be endocytosed in vivo and reach the MHC-II pathway. In the murine tuberculosis model, such LVs induced efficient MHC-II antigenic presentation and triggered both CD8^+^ and CD4^+^ T cells at the systemic and mucosal levels. They also conferred a significant booster effect, consistent with the importance of CD4^+^ T cells for protection against *Mycobacterium tuberculosis*. Given the pivotal role of CD4^+^ T cells in orchestrating innate and adaptive immunity, this strategy could have a broad range of applications in the vaccinology field.

## Introduction

Lentiviral vectors (LVs) provide an efficient vaccine platform due to their strong potential of gene transfer to the nuclei of host cells, including, notably, antigen presenting cells (APCs). Such nuclear transfer of genes initiates the expression of antigens, which readily access the major histocompatibility complex class-I (MHC-I) presentation machinery, i.e., the proteasome, for further triggering of CD8^+^ T cells^1–3^. With the exception of Modified Vaccinia Virus Ankara, able to induce MHC-II-restricted antigen presentation^4^, most viral vectors, including LVs, are relatively ineffective or inoperative in delivering non-secreted antigens to the endosomal MHC-II compartment to trigger CD4^+^ T cells. Although CD8^+^ T cells contribute largely to the immune control of infectious diseases and tumor growth, CD4^+^ T cells are major immune players. In addition to their long lifespan and direct effector functions, CD4^+^ T cells orchestrate the immune system by regulating innate immunity, tailoring B-cell responses and supporting CD8^+^ T-cell effector functions^5^. Therefore, leveraging the potential of LVs to induce CD4^+^ T cells should maximize their success rate in vaccine strategies.

The role of CD4^+^ T cells is notably of critical importance in immune protection against one of the leading causes of death from a single infectious agent, *Mycobacterium tuberculosis* (Mtb), the etiological agent of human pulmonary tuberculosis (TB)^6^. During the chronic stage of infection, this intracellular bacillus is lodged inside phagosomes within infected phagocytes, which results in the presentation of its antigens, essentially by MHC-II molecules. Consequently, it is via MHC-II that adaptive immune effector cells can recognize the infected cells to eradicate them or strengthen their intracellular microbicidal arsenal^7,8^. To develop a poly-antigenic and multistage anti-TB vaccine, we engineered a new generation of LVs that is able to induce MHC-II antigen presentation, resulting in CD4^+^ T-cell initiation.

Collectins are collagen-containing C-type lectins, soluble pattern recognition receptors, that are able to bind to oligosaccharides or lipids at the surface of microorganisms and contribute to their elimination by opsonization or complement activation^9^. Mannan-binding lectin (MBL) or surfactant-associated protein D (SPD) collectins are composed of four distinct segments: (i) an N-terminal cysteine-rich crosslinking domain, (ii) a collagen-like domain, (iii) an α-helical neck domain, and (iv) a carbohydrate-recognition domain (CRD). MBL and SPD can be used as antigen carriers^10^, as their collagen-like domain is permissive to antigen insertion. On the other hand, direct delivery of antigen carriers to APCs via co-stimulatory molecules increases immunogenicity by lowering the amount of antigen required for efficient presentation^11,12^. Therefore, to deliver MBL or SPD antigen carriers to APCs^13–15^, their CRD can be replaced by the ectodomain of the tumor necrosis factor (TNF) family member CD40 ligand (CD40L, CD154)^13,14^. This design was based on the previous demonstration that multimerization of the collectin carriers and the presence of the CD40L ectodomain at their C-terminus are both necessary for the efficient induction of T-cell responses^13,14^.

Taking advantage of the ability of LVs to accommodate large inserts, we developed a new generation of LVs encoding monomers of MBL-CD40L (“M40”) or SPD-CD40L (“S40”) scaffolds harboring multiple Mtb-derived immunogens. Such monomers are theoretically able to spontaneously self-assemble into helicoidal trimers as the first structural units. This potentially leads to a CD40L homo-trimeric configuration, required for CD40 clustering. The trimers can further tetra- or hexamerize to form soluble macromolecule carriers that are able to circulate in biological fluids or be locally taken up by bystander APCs. This suggests that they would likely be taken up by APCs via the endosomal route in vivo, thus reaching the MHC class-II pathway.

As a proof-of-concept, LVs encoding M40 or S40 carrying multiple Mtb immunogens induced efficient MHC-II antigenic presentation in mice and triggered both (poly)functional CD8^+^ and CD4^+^ T cells at both systemic and mucosal levels. By contrast, conventional LVs encoding the same Mtb antigens were unable to induce antigen-specific CD4^+^ T cells. When evaluated in a murine Mtb infection model, one of these new-generation LVs was able to confer a booster protective effect. This approach can be largely extended to LV-based vaccine candidates against numerous other bacterial or viral infectious diseases or cancer, with the critical advantage of inducing robust CD4^+^ T-cell responses, a rare property for a viral-vector vaccine.

## Results

### Design of an LV encoding collectin scaffolds harboring Mtb antigens and a DC-targeting segment

**Fig. 1:**
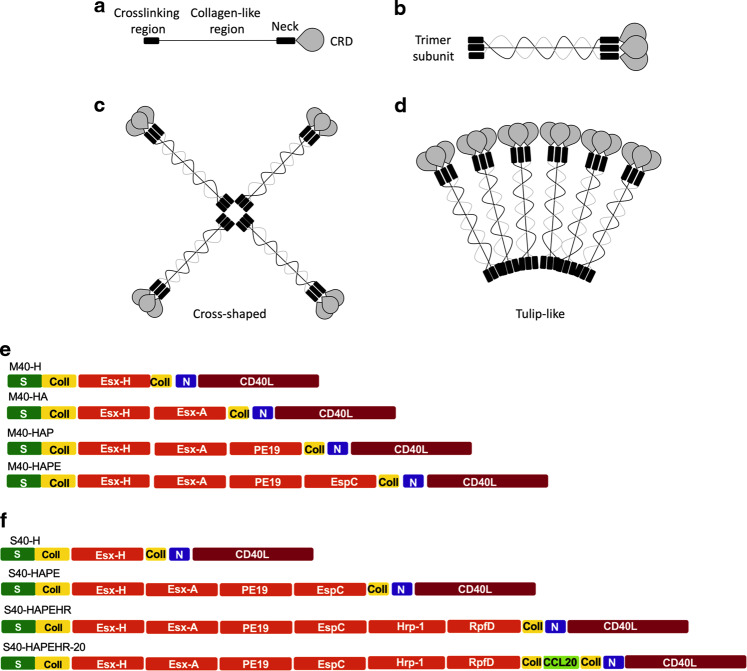
Structure of MBL- and SPD-based antigen carriers. Schematic structure of the MBL and SPD collectin polymers. **a** Structural domains of MBL and SPD. CRD = carbohydrate-recognition domain. **b** MBL and SPD self-assembled collagen-like triple helixes formed by interchain cysteine bonds. **c** SPD cross-shaped dodecamer. **d** SPD and MBL “tulip-bouquet” octodecamers, adapted from^9^. **e**–**f** Schematic representation of the primary structure of the designed M40 (**e**) and S40 (**f**) monomers carrying selected Mtb antigens: crosslinking region (S), collagen-like region (Coll), neck region (N).

MBL and SPD collectins possess four distinct segments: (i) an N-terminal cysteine-rich crosslinking domain, (ii) a collagen-like domain, (iii) an α-helical neck domain, and (iv) a CRD (Fig. 1a). The primary structural MBL or SPD unit consists of self-assembled collagen-like triple helix (Fig. 1b). SPD triple-subunits can tetramerize to form cross-shaped dodecamers (Fig. 1c). SPD or MBL triple-subunits can also hexamerize to form a “tulip-like nano-bouquet” octodecamer (Fig. 1d). The resulting secreted polymers are soluble^9^ and can be used as antigen carriers^13,14^. We generated an LV-based vaccine vector encoding MBL or SPD carriers, potentially usable for an infectious disease controllable by CD4^+^ T cells, using the EsxA, EspC (ESX-1 secretion-associated protein C), EsxH, PE19, hypoxic response protein 1 (Hrp1), and resuscitation promoting factor D (RpfD) immunogens from Mtb^16,17^ (Table S1).

We first engineered the murine MBL to carry the complete sequences of: (i) EsxH alone, (ii) EsxH and EsxA, (iii) EsxH, EsxA, and PE19, or (iv) EsxH, EsxA, PE19, and EspC within the collagen-like region, while replacing its CRD with the murine CD40L_115-260_ ectodomain^13,14^. The prospective MBL polymers will be referred to as “M40-H”, “M40-HA”, “M40-HAP”, and “M40-HAPE”, respectively (Fig. 1e, Table S2, Table S3).

In parallel, we engineered SPD to carry: (i) EsxH alone, (ii) EsxH, EsxA, PE19, and EspC, or (iii) EsxH, EsxA, PE19, EspC, Hrp1, and RpfD_42-154_ within the collagen-like region and substituted its CRD with CD40L_115-260_^13,14^ (Fig. 1f, Table S2, Table S4). The expected SPD polymers are referred to as “S40-H”, “S40-HAPE”, and “S40-HAPEHR”, respectively. To enhance the DC-targeting potential of the resulting fusion protein, we also designed an S40-HAPEHR that carries the murine CCL20_28-97_ segment within the second collagen-like domain (“S40-HAPEHR-20”). CCL20 is the CCR6 ligand, largely involved in the migration and recruitment of immature DCs and lymphocytes^18^.

**Fig. 2:**
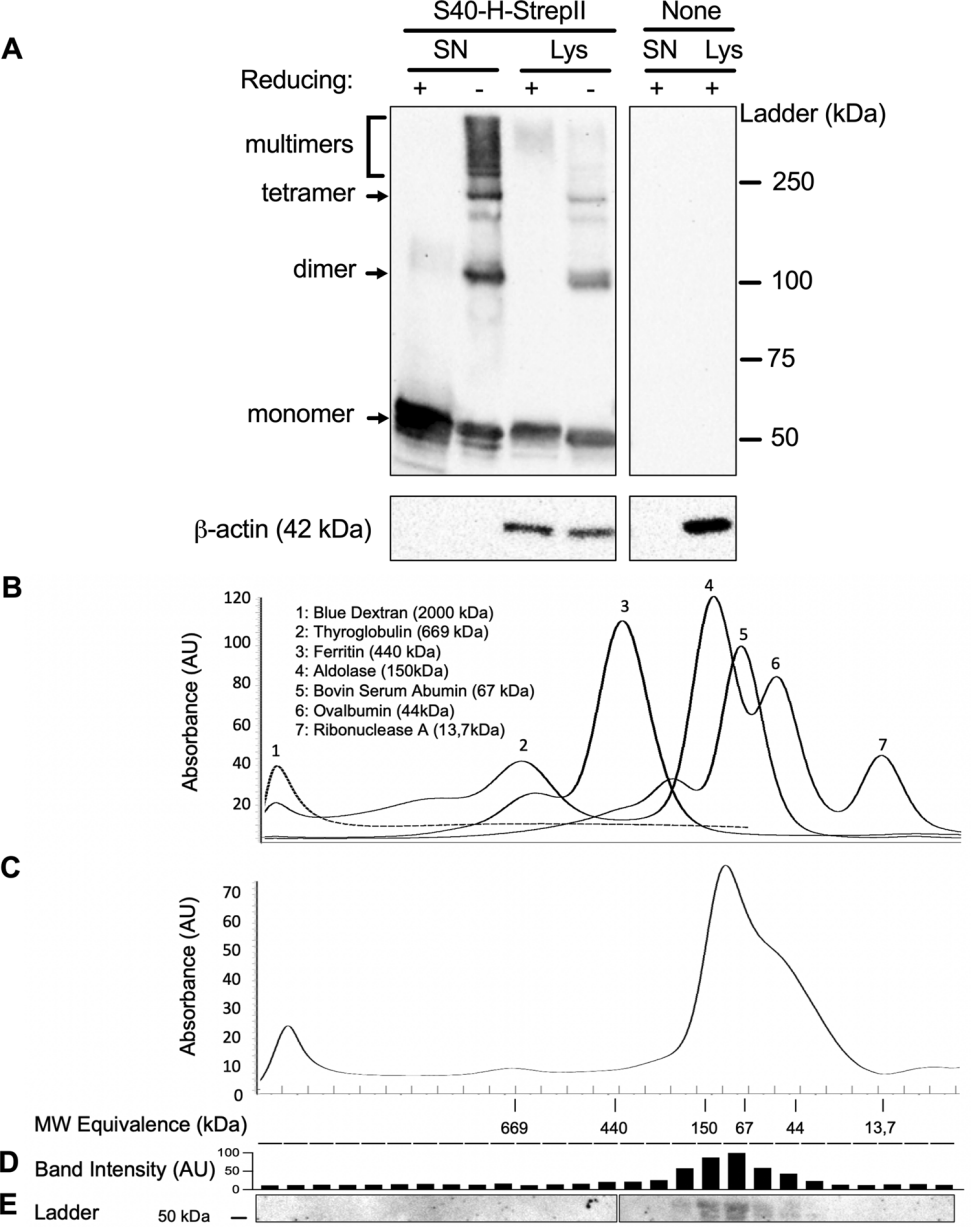
Multimerization of S40-based carriers. **a** Western-blot analysis of supernatants and cell lysates of HEK293T cells, either transduced at an MOI = 100 of LV::S40-H-StrepII or non-transduced, in reducing or non-reducing conditions. β-Actin staining was use of as loading control. **b** Chromatogram of column calibration using a set of purified recombinant proteins on a Superose 6 FPLC column. *X* axis correspond to MW equivalence observed during elution and *Y* axis to UV absorbance (280 nm). **c** Chromatogram of 50x-concentrated supernatant of HEK293T cells transduced with LV::S40-H-StrepII. **d** Quantification of S40-H-StrepII protein by measuring mean pixel intensity of the band of each well, corresponding to the molecular weight of monomeric S40-H-StrepII. **e** Western blot analysis of elution fractions in reducing conditions.

We also generated an LV encoding S40-H StrepII-tagged at its C-terminus (“LV::S40-H-StrepII”) to obtain insights into the polymerization state of antigen-bearing “S40” scaffolds. Supernatants and total lysates from LV::S40-H-StrepII-transduced or non-transduced HEK293T cells were analyzed by SDS-PAGE (Fig. 2a). Under reducing conditions, S40-H monomers were highly detectable in both supernatants and lysates from transduced cells. Under non-reducing conditions, relatively intense bands of S40-H dimers and tetramers and less intense bands corresponding to S40-H trimers were readily detectable in both supernatants and lysates from transduced cells. The lower amount of trimer is obviously related to its further successive multimerization, as the supernatants, and, to a lesser extent, the cell lysates from the transduced cells contained intense bands of high molecular weight. These results show that the engineered recombinant S40 scaffold maintains its multimerization capacity. In order to have a more precise quantification of the high molecular weight species, the multimers of a StrepII-tagged S40-H from a concentrated supernatant of HEK293T transduced cells were fractionated according to their size by exclusion chromatography, then quantified by Western blot (Fig. 2b). While the S40-H-StrepII protein was detected in fractions corresponding to the molecular masses of dimers, trimers and tetramers, it was not possible to detect higher molecular weight multimers. This underlines that the bands of high weight multimer observed in the Western blot are varied but in low amount compared to those of low weight.

### Induction of MHC-II-restricted antigen presentation by LV::M40 and LV::S40

**Fig. 3:**
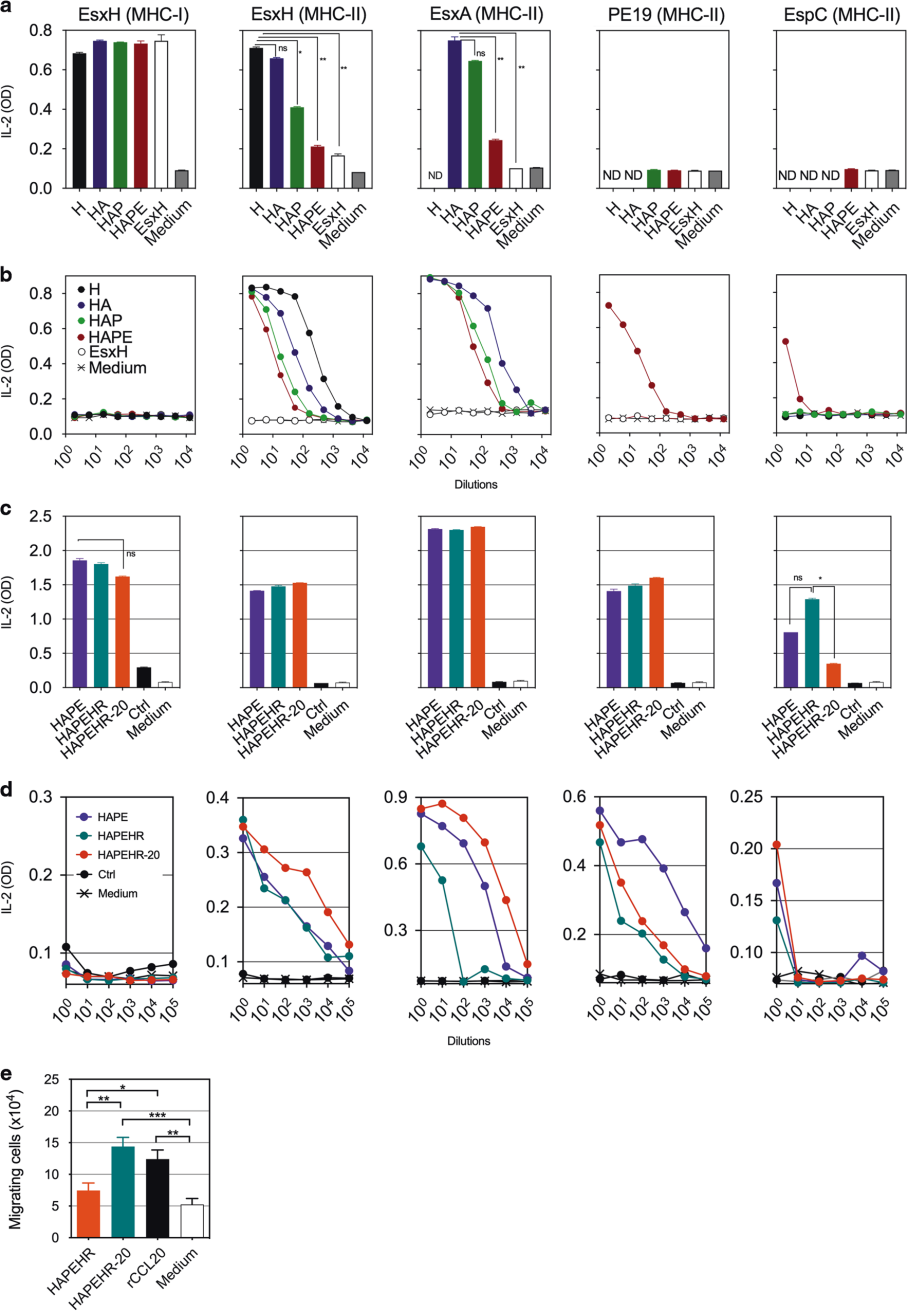
Properties of backbones at recruiting APC and inducing antigenic presentation by both the MHC-I and-II pathways. **a**, **c** BM-DCs from BALB/c (H-2^d^) or C57BL/6 (H-2^b^) mice were transduced (MOI = 20) with LV::M40-H, -HA, -HAP, or -HAPE (**a**) or LV::S40-H, -HAPEHR, or -HAPEHR-20 (**c**) under the transcriptional control of the BCUAG promoter. Control cells were transduced with LV::EsxH alone. **b**, **d** BM-DCs from BALB/c or C57BL/6 mice were incubated with successive dilutions of supernatants of HEK-293T cells transduced (MOI = 20) for 48 h with each of the indicated LVs. On day 3 after addition of the LVs or day 1 after incubation with the HEK293T cell supernatants, the presentation of MHC-I- or -II-restricted epitopes of the EsxH, EsxA, PE19, or EspC mycobacterial antigens by DCs was assessed by their co-culture with T-cell hybridomas specific for EsxH:20-28 (YB8 cell line, restricted by K^d^), EsxH:74-88 (1G1 cell line, restricted by I-A^d^), EsxA:1-20 (NB11 cell line, restricted by I-A^b^), PE:19:1-18 (IF6 cell line, restricted by I-A^b^), or EspC:45:54 (IF1 cell line, restricted by I-A^b^). Results are presented as the concentration of IL-2 produced by the T-cell hybridomas 24 h after the beginning of the co-cultures. The amount of IL-2 found in the co-culture supernatants is proportional to the efficacy of antigenic presentation by DCs and TCR triggering. This assay does not measure the physiological response induced in the T cells but only provides an indicator of the stimulation of the T-cell hybridoma TCR. **e** Impact of HAPEHR and HAPEHR-20 proteins on migration of BM-DCs in a transwell system. Supernatant of HEK293T cells transduced (MOI = 100) with LV::S40-HAPEHR or LV::S40-HAPEHR-20 (*n* = 6). Statistical significance was evaluated using the Mann–Whitney test (* = *p* < 0.05, ** = *p* < 0.01, *** = *p* < 0.001, ns non-significant).

DCs (H-2^d^ or H-2^b^) were directly transduced with LV::M40-H, -HA, -HAP, or -HAPE using a composite β2m-CMV promoter (“BCUAG”) comprised of the human β2-microglobulin (β2 m) promoter^19^ and the human cytomegalovirus immediate early enhancer and promoter (CMV)^20^ (Fig. S1A, B). Control DCs were transduced with a conventional LV encoding EsxH under the same promoter, without insertion into an engineered scaffold. Three days post-transduction, the DCs were co-cultured with T-cell hybridomas specific for the immunodominant epitopes of each of the Mtb antigens. The DCs transduced with either LV were largely able to induce the presentation of EsxH via MHC-I (Fig. 3a). In contrast to the conventional LV::EsxH, the LV encoding M40-H, -HA, -HAP, or -HAPE induced MHC-II-restricted presentation of EsxH and EsxA when these antigens were included. No MHC-II presentation of PE19 or EspC was detected in this context, which, in the case of EspC, can be explained by the relatively weak sensitivity of the T-cell hybridoma (Fig. S2). We evaluated whether M40 or S40 carriers secreted by transduced cells induce presentation through MHC-II by incubating DCs with successive dilutions of M40-H-, -HA-, -HAP-, or -HAPE-containing supernatants from HEK293T cells transduced at a multiplicity of infection (MOI) of 1 of the corresponding LVs (Fig. 3b). On day 1 after incubation, co-culture of the DCs with T-cell hybridomas showed the DCs to be unable to present EsxH via MHC-I, strongly suggesting that endocytosis/micropinocytosis or CD40-mediated cell entry of the M40 carrier does not allow their access to the MHC-I machinery, in contrast to observations made by others^21^. In net contrast to MHC-I, DCs incubated with M40-H-, -HA-, -HAP-, or -HAPE-containing supernatants were highly efficient at inducing presentation of the respective antigens via MHC-II, including EspC. The level of antigen presentation tended to decrease with a growing number of antigens carried by the M40 scaffold (Fig. 3a, b). In a mutually non-exclusive manner, this may result from: (i) slight structural instability of the carriers with the insertion of an increasing number of antigens or (ii) competition among the multiple T-cell epitopes for the available MHC presentation sites.

Direct transduction of DCs with LV::S40-HAPE, -HAPEHR or -HAPEHR-20 also induced efficient MHC-I- and -II-restricted presentation of the selected Mtb antigens (Fig. 3c). Incubation of DCs with successive dilutions of supernatants from HEK293T cells transduced at an MOI of 1 with LV::S40-HAPE, -HAPEHR, or -HAPEHR-20 induced MHC-II-restricted presentation of the Mtb antigens (Fig. 3d). In the absence of identified T-cell epitopes and T-cell hybridomas specific to Hrp1 and RpfD, we studied the immunogenicity of these antigens in the context of the developed vectors in vivo, as detailed below. Insertion of 4–6 Mtb antigens to the S40 carrier gave the highest levels of antigenic presentation via MHC-II by DCs directly transduced with LV::S40 (Fig. 3c). In addition, in contrast to the results obtained with M40 (Fig. 3b), the supernatants from HEK293T cells transduced at the same MOI with various LV::S40 constructs with an increasing number of Mtb antigens did not show a progressive decrease in the efficiency of antigen presentation via MHC-II (Fig. 3d). Assays performed with DCs incubated with synthetic peptides for the homologous T-cell epitopes showed the same sensitivity as the T-cell hybridoma-based presentation assay (Fig. S2). At last, impact of the insertion of the CCL20 protein sequence into the HAPEHR backbone was evaluated by use of supernatants from HEK293T cells transduced with LV::S40-HAPEHR or LV::S40-HAPEHR-20 in a transwell assay. This experiment demonstrated an improved ability of HAPEHR-20, compared to the native HAPEHR, to mobilize bone-marrow derived DCs in vitro (Fig. 3e).

These results show that, in net opposition to conventional LVs, this new generation of LVs encoding secreted scaffolds that can incorporate numerous antigens and immune mediators possess a strong capacity to induce MHC-II-restricted antigen presentation and thus provide a valuable platform for both CD4^+^ and CD8^+^ T-cell induction.

### The potential of M40 and S40 to induce DC maturation

**Fig. 4:**
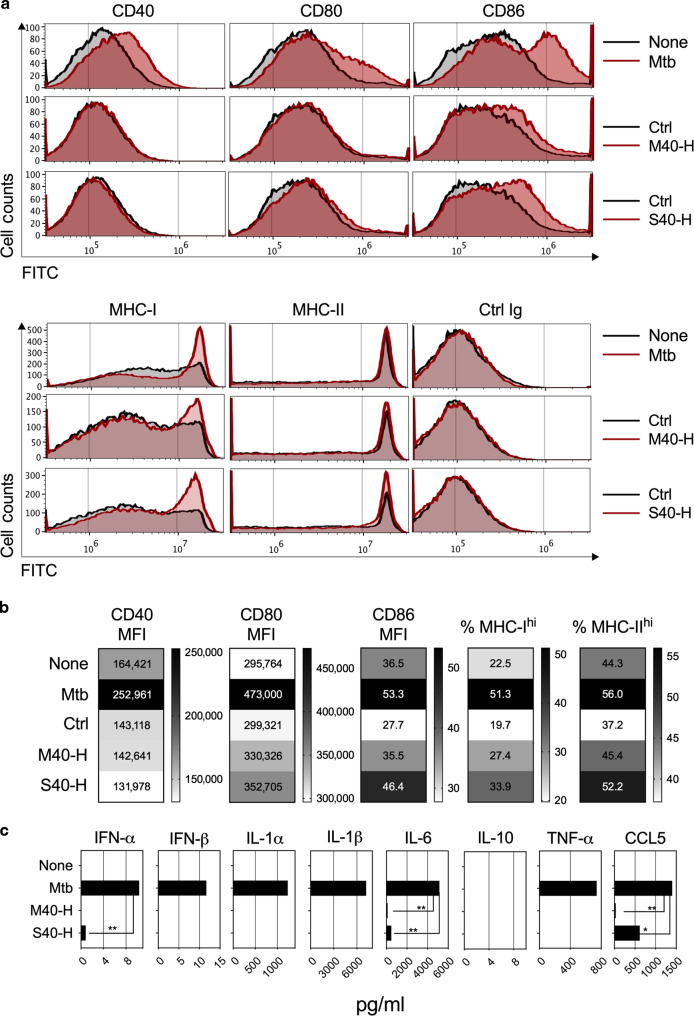
Phenotypic Maturation of DCs Induced by M40 or S40. **a** Phenotypic maturation of BM-DCs from C57BL/6 mice infected at an MOI of 5 with Mtb, as a positive control, or incubated with supernatants from HEK-293T cells transduced (MOI = 20) with LV::EsxH alone (Ctrl), LV::M40-H, or LV::S40-H. Expression of co-stimulatory or MHC molecules on the surface of CD11b^+^ CD11c^+^ cells at 24 h post-immunization was assessed by cytometry. **b** Heatmaps showing the mean fluorescence intensity (MFI) of CD40 and CD80 surface expression and the percentage of CD86^hi^, MHC-I ^hi^, and MHC-II ^hi^ DCs. **c** Quantitation of inflammatory mediators in the culture supernatants of the same DCs. Results are representative of at least two independent experiments.

We evaluated the potential of M40 and S40 carriers to induce DC maturation by incubating BM-DCs with supernatants from HEK293T cells transduced at an MOI of 1 with LV::M40-H or LV::S40-H. In parallel, DCs were incubated with supernatants from HEK293T cells transduced with the conventional LV::H, as a negative control, or infected with Mtb, as a positive control. The surface expression of co-stimulatory and MHC molecules was assessed for CD11b^+^ CD11c^+^ cells on day 1 post incubation (Fig. 4a, b). We detected no increase in CD40 surface expression for DCs incubated with M40-H or S40-H, likely due to the direct interaction of CD40 with M40-H or S40-H (Fig. 4a, b). CD80 upregulation was only detected for DCs incubated with S40-H, whereas CD86 upregulation and an increase in the percentage of MHC-I^hi^ and MHC-II^hi^ cells was observed for DCs incubated with M40-H or S40-H. Very slight functional maturation was also induced by S40-H, as shown by the secretion of minute levels of IFN-α, IL-6, and CCL5, but not IFN-β, IL-1α, IL-1β, IL-10, or TNF-α, detected by a multiplex ELISA assay applied to the supernatants of the same DCs (Fig. 4c). Therefore, through the induction of M40 or S40 secretion, this new generation of LVs is able to induce a certain degree of DC maturation, which is instrumental for appropriate T-cell activation. Taken together, these results indicated that soluble S40-H resulted in a higher degree of DC phenotypic and functional maturation than M40-H (Fig. 4).

### T-cell immunogenicity of LVs encoding M40 or S40 carrying a single or multiple Mtb immunogens

We next assessed the immunogenicity of this new generation of LVs. BALB/c mice (*n* = 3/group) were immunized s.c. with LV::M40-H carrying the β2 m^22^, CMV^20^, or composite BCUAG promoter (Fig. S1) to obtain insights on the possible consequences of distinct antigen carrier transcription profiles on the induction of immune responses. Control mice were immunized s.c. with a conventional LV::EsxH. On day 13 post injection (dpi), the splenocytes from immunized mice were stimulated with EsxH:20–28 (MHC-I) or EsxH:74–88 (MHC-II) peptides^23,24^. Although the conventional LV::EsxH induced antigen-specific MHC-I-restricted CD8^+^ T cells, it was unable to induce antigen-specific MHC-II-restricted CD4^+^ T cells, as detected by ELISPOT (Fig. S3). The LV::M40-H vectors all induced both CD8^+^ T and CD4^+^ T cells (Fig. S3). Intracellular cytokine staining (ICS) showed the multifunctional properties of these CD8^+^ and CD4^+^ (Fig. S4A, B) T cells. Functional CD8^+^ T cell effectors were mainly distributed among IFN-γ^+^ (single positive), IFN-γ^+^ TNF-α^+^ (double-positive), and IFN-γ^+^ TNF-α^+^ IL-2^+^ (triple positive) subsets, whereas CD4^+^ T cells were essentially IFN-γ^+^ (single positive) or IFN-γ^+^ TNF-α^+^ IL-2^+^ (triple positive). In contrast to CD8^+^ T-cell responses, no EsxH-specific CD4^+^ T-cell responses were detected in the mice immunized with the conventional LV::EsxH. No consistent quantitative or qualitative differences were detected in the T-cell responses in mice immunized with LV::M40 carrying each of the distinct promoters.

We evaluated the immunogenic potential of the developed poly-antigenic LV::M40-HAPE by immunizing C57BL/6 mice (*n* = 3/group) s.c. with LV::M40-HAPE carrying the β2 m, CMV, or BCUAG promoter. On 14 dpi, CD8^+^ and CD4^+^ T-splenocyte responses specific to EsxH:3–11 (MHC-I), EsxA:1-20 (MHC-II), PE19:1-18 (MHC-II), or EspC:45-54 (MHC-I and -II)^25^ were detected in all mice, as assessed by ELISPOT (Fig. S5). The conventional LV::HAPE induced CD8^+^, but not CD4^+^, T cells. ICS analysis of the splenocytes from the same mice showed the multifunctional properties of the induced CD8^+^ (Fig. S6A, C) and CD4^+^ (Fig. S6B, D) T cells. Functional CD8^+^ T cell effectors were again mainly distributed among IFN-γ^+^ single-positive, IFN-γ^+^ TNF-α^+^ double-positive, and IFN-γ^+^ TNF-α^+^ IL-2^+^ triple-positive subsets. CD4^+^ T cells specific to EsxA, PE10, or EspC antigen were preferentially distributed among IFN-γ^+^ single-positive, IFN-γ^+^ TNF-α^+^ double-positive, and or IFN-γ^+^ TNF-α^+^ IL-2^+^ triple-positive subsets (Fig. S6D). No consistent quantitative or qualitative differences were detected in the T-cell responses in mice immunized with LV::M40-HAPE carrying each of the distinct promoters. Again, in contrast to CD8^+^ T-cell responses, no antigen-specific CD4^+^ T-cell responses were detected in the mice immunized with the conventional LV::HAPE.

**Fig. 5:**
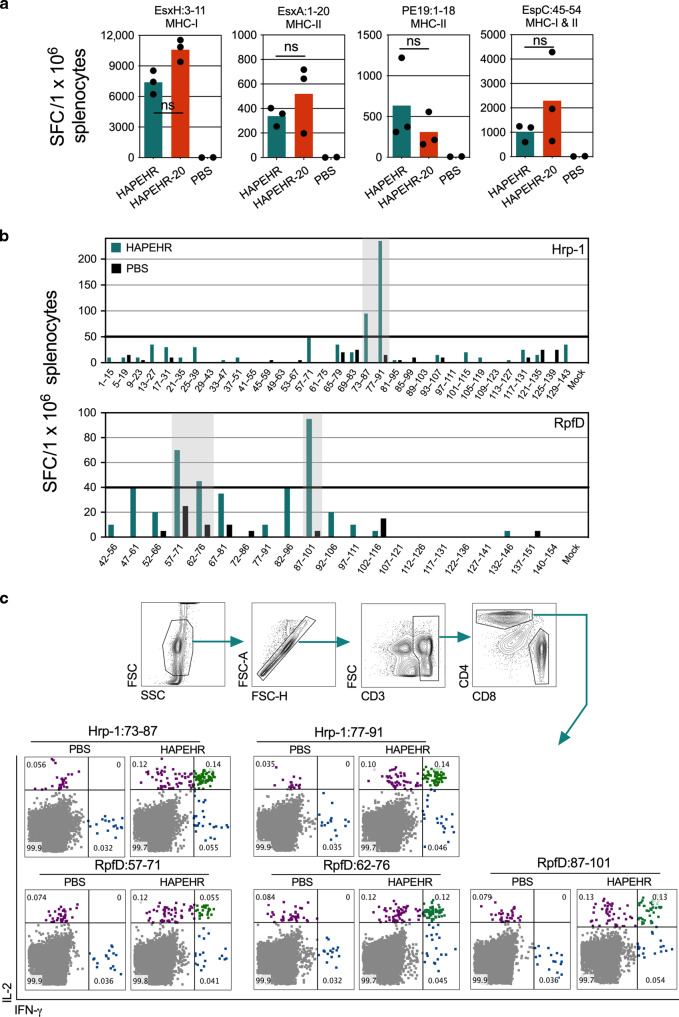
Immunogenicity of Multi-Antigenic LV::S40-HAPEHR or LV::S40-HAPEHR-20. **a** IFN-γ T-cell responses, as assessed by ELISPOT on day 13 post-immunization, in the spleens of individual C57BL/6 mice (*n* = 3) immunized s.c. with 1 × 10^8^ TU/mouse of LV::S40-HAPEHR or LV::S40-HAPEHR-20. The frequency of responding T cells was determined following in vitro stimulation with the indicated synthetic peptides. Quantitative differences between the groups of mice immunized with LV::S40-HAPEHR or LV::S40-HAPEHR-20, were not statistically significant (Mann–Whitney test). **b** Epitope mapping of Hrp-1 and RfpD, as determined using pooled splenocytes from 3 C57BL/6 mice/group injected with PBS or immunized s.c. with 1 × 10^8^ TU/mouse of LV::S40-HAPEHR prior to stimulation with each of the individual peptides from the Hrp-1- or RpfD-derived overlapping 15-mers offset by five amino acids. **c** Cytometric analysis of intracellular IFN-γ vs IL-2 staining of CD4^+^ T splenocytes after stimulation with 10 µg/ml of the indicated peptides encompassing the immunodominant epitopes identified in **b**. Pooled splenocytes from three mice/group were used.

We then focused on the immunogenicity of LV::S40 vectors. We established the induction of both CD8^+^ and CD4^+^ T cells specific to EsxH, EsxA, PE19, and EspC in C57BL/6 mice (*n* = 3/group) immunized s.c. with LV::S40-HAPEHR or LV::S40-HAPEHR-20 (Fig. 5a). The immunogenicity of Hrp-1 and RpfD was assessed by mapping their epitopes using splenocytes from LV::S40-HAPEHR-immunized mice by ELISPOT (Fig. 5b). The Hrp-1:77-91 (SIYYVDANASIQEML), RpfD:57-71 (IAQCESGGNWAANT), and RpfD:87-101 (SNGGVGSPAAASPQQ) immunogenic regions were identified. Although these epitopes did not induce CD8^+^ T cells (not shown), they did trigger a CD4^+^ T-cell response, as assessed by ICS (Fig. 5c). Overall, these results provide evidence of the induction of robust, polyfunctional CD4^+^ T-cell responses by immunization with this new generation of LVs.

Given the better results of: (i) antigenic presentation (Fig. 3), (ii) DC maturation (Fig. 4), and (iii) CD4^+^ T-cell immunogenicity against six Mtb antigens provided by LV::S40 vectors (Fig. 5), we chose to continue with S40 carrier for the remainder of the study.

### Immunogenicity of the poly-antigenic multistage LV::S40 at the mucosal level

**Fig. 6:**
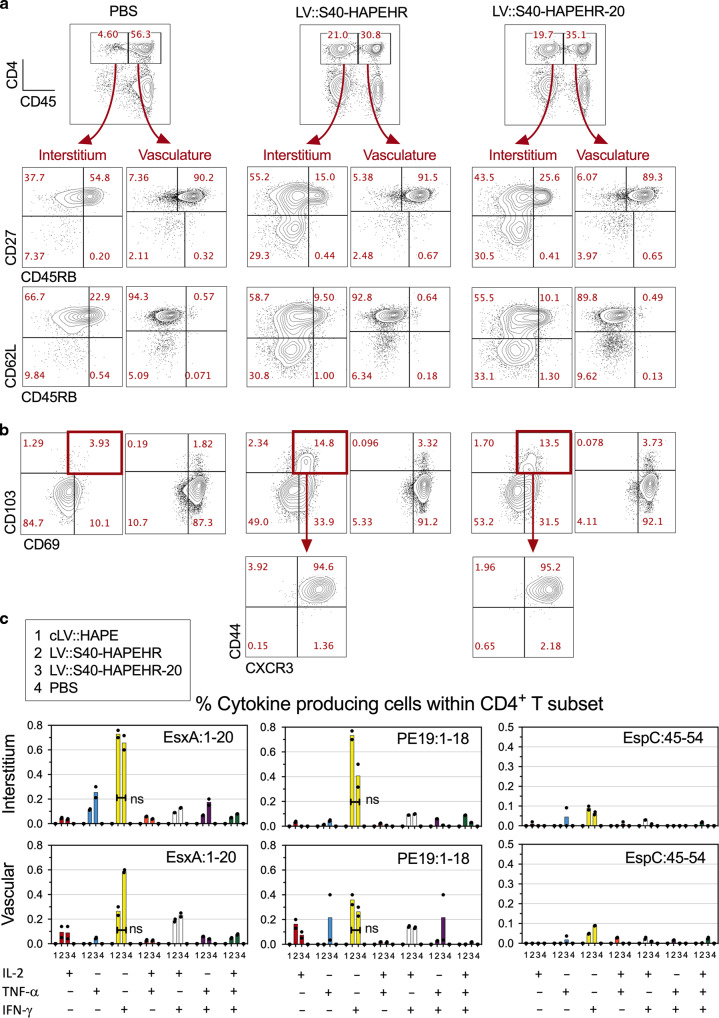
Features of mucosal CD4^+^ T cells triggered by i.n. immunization with LV::S40-HAPEHR or LV::S40-HAPEHR-20. C57BL/6 mice (*n* = 3/group) were immunized i.n. with 1 × 10^8^ TU of LV::S40-HAPEHR or LV::S40-HAPEHR-20. On 14 dpi, lung CD4^+^ T cells were distinguished by their location within the interstitium (CD45_i.v_^−^) or vasculature (CD45_i.v_^+^) by an i.v. injection of PE-anti-CD45 mAb. **a** Profile of CD27 vs CD62L or CD45RB, and **b** CD103 vs CD69, or CD44 vs CXCR3 of lung CD4^+^ T cells of the interstitium or vasculature. **c** Percentage of (poly)functional CD4^+^ T cells specific to EsxA, PE19, or EspC in the lung interstitium or vasculature, as determined by ICS. Results, representative of two independent experiments, were obtained using pooled lungs for each group (*n* = 3/group). Individual points represent technical duplicates. Results are representative of at least three independent experiments.

**Fig. 7:**
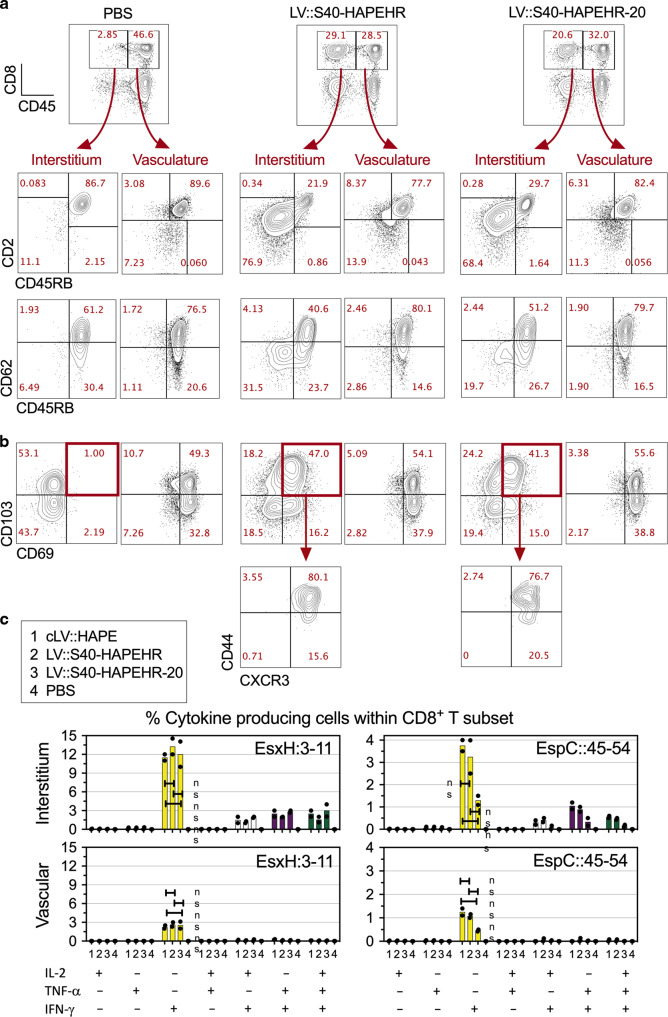
Features of mucosal CD8^+^ T cells induced by i.n. immunization with LV::S40-HAPEHR or LV::S40-HAPEHR-20. The immunized C57BL/6 mice are those studied in the Fig. 6. **a** Shown are lung CD8^+^ T cells, distinguished for their location within the interstitium (CD45_i.v_^−^) or in the vasculature (CD45_i.v_^+^). Profile of CD27 vs CD62L or CD45RB, and **b** CD103 vs CD69, or CD44 vs CXCR3 of the lung CD8^+^ T cells from the interstitium or vasculature. **c** Recapitulative percentages of (poly)functional CD8^+^ T cells specific to EsxH or EspC in the lung interstitium (top) or vasculature (bottom), as determined by ICS. Results are representative of at least two independent experiments.

We next evaluated the immunogenicity of LV::S40-HAPEHR and LV::S40-HAPEHR20 in C57BL/6 mice immunized intranasally (i.n.) with 1 × 10^8^ TU. Intravenous (i.v.) injection of the immunized mice 14 dpi with PE-anti-CD45 mAb 3 min before sacrifice showed massive T-cell recruitment to the lung interstitium distinct from that in the vasculature^26^. The lung interstitial (CD45_i.v.−_) CD4^+^ (Fig. 6a) or CD8^+^ (Fig. 7a) T cells of LV::S40-HAPEHR- or LV::S40-HAPEHR20-vaccinated mice contained an elevated number of CD27^−^ CD45RB^−^ CD62L^−^ migrant effectors and CD69^+^ CD103^+^ resident cells relative to their PBS-injected counterparts (Fig. 6b, Fig. 7b). Most of the CD69^+^ CD103^+^ CD4^+^ or CD8^+^ T cells were CD44^+^ CXCR3^+^. Some degrees of CD4^+^ and CD8^+^ T-cell recruitment to the lungs were also detected with a conventional LV::HAPE (cLV), compared to PBS, albeit the percentages of interstitial CD69^+^ CD103^+^ inside the CD4^+^ subset (Fig. S7A), or those of interstitial CD27^−^ CD45RB^-^ activated cells inside the CD8^+^ subset (Fig. S7B) in cLV-treated mice did not reached those in LV::S40-HAPEHR- or LV::S40-HAPEHR20-immunized mice (Fig. 6b and Fig. 7a). ICS analysis of these cells showed the presence of (poly)functional CD4^+^ (Fig. 6c) and CD8^+^ (Fig. 7c) antigen-specific T cells, essentially located in the lung interstitium. cLV administered i.n. induced mucosal antigen-specific CD8^+^ (Fig. 7c), but not mucosal CD4^+^ (Fig. 6c), T cells. We also thoroughly investigated the impact of the i.n. administration of LV::S40-HAPEHR or LV::S40-HAPEHR20 on the composition of the lung innate immune cell subsets on day 1 (Fig. S8A) and day 2 (not shown) post injection. We detected no significant differences in the proportions of various cell subsets vs total lung CD45^+^ cells relative to PBS-treated mice (Fig. S8B). This observation demonstrates that, despite the capacity of the developed vectors to induce robust T-cell immunity, their administration via the mucosal route has no notable impact on the features of mucosal innate immune cells, indicating the absence of an adverse inflammatory effect after mucosal administration of these vectors.

Although LV::S40-HAPEHR and LV::S40-HAPEHR20 displayed similar efficiency in inducing T cells, based on the capacity of LV::S40-HAPEHR20 to better mobilize DCs in vitro (Fig. 3e), for further experiments we continued with LV::S40-HAPEHR20 vector.

### Protective effect of an LV::S40-HAPEHR-20 boost against Mtb infection

**Fig. 8:**
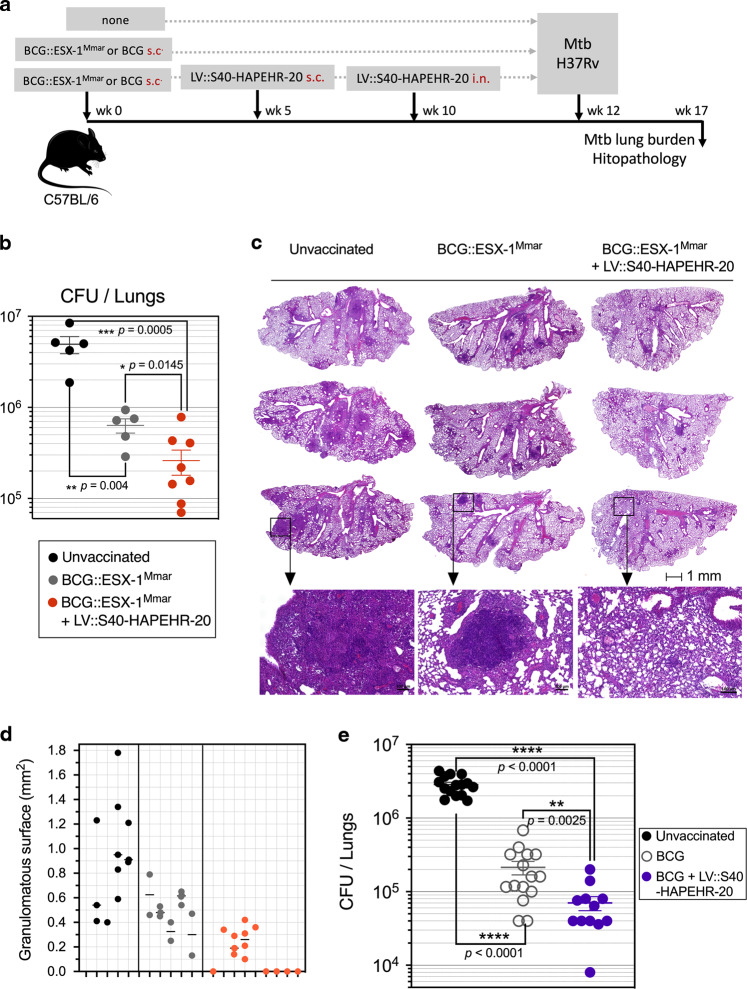
Protective potential of an optimized poly-antigenic LV as a booster against Mtb. **a** Timeline of the prime-boost-challenge performed in C57BL/6 mice (*n* = 5–8 mice/group). **b** Mtb burden as quantitated by CFU counting in the lungs of BCG::ESX-1^Mmar^-primed and LV::S40-HAPEHR-20-boosted mice on week 5 post challenge. **c** Whole-lung section of the left lobe and **d** quantification of the number and size of lung granulomatous lesions per mouse in each experimental group. **e** Mtb burden quantified by CFU counting in the lungs of BCG-primed and LV::S40-HAPEHR-20-boosted C57BL/6 mice following the timeline indicated in **a**, but using Danish BCG for the prime immunization and i.n. Mtb challenge. The pooled results from two independent experiments are shown (*n* = 6–8 mice/group in each experiment). The significance of the differences was determined using the Mann–Whitney test. ns not significant.

Prime-boost strategies using BCG or an improved live-attenuated vaccine for priming and subunit vaccine candidates for boosting is a promising approach to improve the incomplete efficiency of BCG. We assessed the booster potential of LV::S40-HAPEHR-20 by immunizing C57BL/6 mice s.c. at week 0 with 1 × 10^6^ CFU of a genetically improved BCG, i.e., BCG::ESX-1^Mmar^ vaccine candidate^27^ or leaving them unvaccinated (Fig. 8a). This approach provided the opportunity to perform a prime-boost with the developed LV vaccine, as this live-attenuated vaccine actively secretes EsxA and EspC. A group of BCG::ESX-1^Mmar^-primed mice was boosted s.c. with 1 × 10^8^ TU of LV::S40-HAPEHR-20 on week 5 and then again boosted i.n. on week 10 with the same LV to recruit the induced immune effectors to the lung mucosa. The choice of the i.n. route of the last boost, after a primary immunization by the systemic route, was based on our observations in the fields of Mtb^11,28^ and SARS-CoV-2^22,29,30^ and those of many other studies^31^ showing the importance of boost immunization by the mucosal i.n. route in the immune control of respiratory pathogens. The aim was to target the immune arsenal toward the primary site of infection. On week 12, mice were challenged with ≈200 CFU of Mtb H37Rv via aerosol and lung mycobacterial burdens were determined on week 17 (Fig. 8b). The average lung Mtb load in the primed-boosted mice was ≈2.5 log_10_ lower that of unvaccinated controls (Mann–Whitney test, *p* = 0.0005) and ≈1 log_10_ lower than that of their BCG::ESX-1^Mmar^-vaccinated counterparts (Mann–Whitney test, *p* = 0.0415).

Granulomas were present in all non-vaccinated and BCG::ESX-1^Mmar^-vaccinated animals but in only 4/9 primed and boosted mice (Fig. 8c, d). They consisted primarily of lymphocytes and epithelioid or foamy macrophages. Polynuclear cells were rare. In non-vaccinated animals, the granulomas were diffuse and disorganized, with a tendency to fuse to each other. In the lungs of vaccinated animals, the granuloma were smaller and neatly delimited from the rest of the parenchyma and assumed a lymphocyte-inside/macrophage-outside organization. Remarkably, five mice in the primed-boosted group had no detectable granulomatous lesions. All histology preparations exhibited at least some degree of alveo-interstitial inflammatory lesions (Fig. 8c), characterized by a mononuclear cell infiltrate, sometimes interspersed with polynuclear cells. In vaccinated mice, the interstitial involvement was generally mild. Infiltrated alveoli were present throughout the sections and were generally separated by free alveoli, although in some preparations, pre-consolidation areas of limited size were observed.

Following the same prime-boost timeline (Fig. 8a), but using Danish BCG for the prime instead of BCG::ESX-1^Mmar^ and i.n. instead of aerosol Mtb challenge, the largely significant protective booster effect of LV::S40-HAPEHR-20 was also confirmed in the lungs of C57BL/6 mice (Mann–Whitney test, *p* = 0.0025) (Fig. 8e). These results reinforce the interest of this optimized LV in inducing or boosting T-cell immunity against infectious diseases.

## Discussion

We have developed a new generation of multifunctional LVs which, relative to the conventional vector, has been altered to: (i) facilitate poly-antigen delivery, (ii) target antigens to the APCs that it activates, (iii) route antigens through the MHC-II pathway, and (iv) induce, in addition to CD8^+^ T cells, robust and polyfunctional CD4^+^ T-cell responses. Such LVs were tailored to induce the secretion of multimeric protein carriers formed by truncated collectin-based scaffolds capable of carrying several antigens, as well as protein components with adjuvant or chemo-attractant properties. This was achieved by the insertion of potent immunogens within the collagen-rich regions of MBL or SPD, substituted with the CD40L ectodomain in their CRD region. Self-assembly and polymerization of the monomers produced in the LV-transduced cells in vitro or in vivo resulted in secreted multimeric carriers capable of interacting with CD40^+^ cells, including APCs and, notably, DCs. It is known that antigen delivery to appropriate surface receptors of DCs improves the efficacy of antigenic presentation by several orders of magnitude^11,32,33^. The conventional LV per se, as prepared under our conditions, was barely inflammatory and induced almost no DC phenotypic or functional maturation, even when used at very high doses. The capacity of LVs to transiently induce the secretion of minute levels of IFN-I in vivo and IFN-I signaling in DCs in vivo was not linked to their strong T-cell immunogenicity^25^. Unlike conventional LVs, the new generation of LVs described here induced a certain degree of DC maturation, most likely via CD40 clustering by the trimeric extremities of the M40 and S40 carriers. Therefore, these vectors assemble (i) the intrinsic and outstanding CD8^+^ T-cell immunogenicity of conventional LVs and (ii) the properties of slight adjuvantation, antigen delivery to DC surface receptors, antigen routing to MHC-II, and CD4^+^ T-cell immunogenicity of the secreted multimeric scaffolds that they encode. One can ask whether multimerization of the carriers was required to induce DC maturation. However, the study of DC maturation by various biochemically isolated multimers, which are difficult to protect from contamination by endotoxins, would have been technically very challenging. The fact that antigen carriers based on the structure of collectins multimerized and fused with the CD40L ectodomain induce DC activation, had been also previously well established^13,14^. The degree of DC maturation induced by M40 and S40 was relatively low, but sufficient to induce T-cell responses^19,34,35^. Higher DC-activating potential could, on the other hand, be considered to be too inflammatory, which would be an obstacle to their use in vaccination, notably, via the mucosal route.

As TB is a disease primarily controlled by CD4^+^ T-cell responses, as a first application, we investigated these optimized LVs for their potential in inducing T-cell responses against selected Mtb antigens with preferential expression at distinct infection phases. The EsxA and EspC virulence factors are strongly immunogenic due to their small size and their active secretion through the ESX-1 type VII secretion system (T7SS), which favors their access to MHC presentation machineries of the host phagocytes^16,17^. The highly immunogenic EsxH^23,24,36^, secreted through the ESX-3 T7SS, has shown protective potential in several subunit vaccine candidates. EsxH and its close relative EsxR are present in all Mtb clinical isolates thus far studied^37^. The inclusion of PE19 from the large family of PE/PPE Mtb proteins, secreted through ESX-5 T7SS^17,38–43^, was based on its content in T-cell epitopes, shared by numerous homologous members of the large PE multigenic family, with various expression profiles at distinct stages of infection, which may generate a constant display of such shared T-cell epitopes throughout infection^40,43–45^. As Mtb evolves from an acute to persistent phase, the *dosRS* two-component regulatory system initiates the transcription of 48 genes, including *rv2626c*^46,47^. The resulting Hrp1 can be a target of host adaptive immunity when the bacilli are quiescent within the granuloma^48^. In addition, Mtb possesses five resuscitation promoting factors (RpfA-E) which contribute to the cell-wall remodeling during bacterial division, at both the acute and reactivation phases^49–51^. We selected RpfD because of its demonstrated immunogenicity in both mice^52^ and humans with latent TB^53^.

Here, we demonstrate in vitro the property of LV::M40 and LV::S40, as well as the M40 and S40 carriers secreted by LV::M40- and LV::S40-transduced cells, to induce MHC-II-restricted presentation of the Mtb antigens inserted within their collagen-like domains. One can ask whether conventional LV-EsxH leads to the expression of secreted EsxH which could contribute to DC maturation. The EsxH protein does not have a signal sequence for secretion by transduced host cells. It is secreted in the physiological context of Mtb via the ESX-3 T7SS^16,17^. Therefore, the likelihood that EsxH could be secreted by the transduced cells is very low, which is consistent with the lack of MHC-II-restricted presentation of EsxH by DCs incubated with supernatants from HEK293T cells transduced with conventional LV::EsxH.

We show, in vivo, efficient induction of both (poly)functional CD8^+^ and CD4^+^ T-cell effectors at both the systemic and mucosal level following s.c. or i.n. immunization with LV::M40 or LV::S40. Notably, only one i.n. immunization was sufficient to generate high-quality CD8^+^ and CD4^+^ T-cell effectors, with activated/effector/resident memory phenotypes and localized to the pulmonary interstitium. In future studies, it will be informative to determine whether such T cells are located in the lung tertiary lymphoid organs^54^.

One of the multimeric carriers, S40-HAPEHR also included a segment of CCL20, a strong chemo-attracting chemokine. The receptor for CCL20, CCR6, is expressed on lymphocytes and DCs^55^. We showed in vitro that inclusion of CCL20 into S40-HAPEHR reinforced the migration of DCs. Prime immunization with improved live-attenuated vaccine candidates and boosting with subunit vaccines is a promising approach. We used LV::S40-HAPEHR-20 as a subunit booster in the mouse TB model after prime immunization with either the BCG Danish or BCG::ESX-1^Mmar^ vaccine candidates, with improved protective potential relative to the parental BCG^27^. Indeed, the lung Mtb burden was statistically reduced by ~1 log_10_ after LV::S40-HAPEHR-20 boosting. One limitation of our study was not having a comparison with a conventional LV::HAPE and LV::S40-HAPEHR-20 in parallel in the protection experiment. This was based on the inefficiency of conventional LV in inducing antigenic presentation via MHC-II and CD4^+^ T responses which are the best correlates of protection against Mtb. In addition, and importantly, multiplying the number of experimental groups to include more controls is technically difficult, in particular, when the Mtb challenge is performed by the aerosol route. Indeed, our aerosol device does not allow inclusion of more than 20 mice per infection cycle. The Mtb input was relatively variable between aerosol infection cycles, making the analyses complex. The study of protection against Mtb using the developed LV will then be extended to alternative protocols in terms of vaccination regimen, doses and routes of administration used for prime and boost and to more antigenic designs for further comparisons, that are beyond the scope of this study.

A plasmid DNA encoding SPD-CD40L has already been used as an adjuvant when mixed with another plasmid DNA encoding the HIV-1 Gag protein and led to significant enhancement of CD8^+^ T cell responses^13^. In net contrast to the LV platform developed here, this plasmid adjuvant was unable to induce a CD4^+^ T-cell proliferative or cytokine response. Insertion of SPD-Gag-CD40L into adenoviral vector serotype 5 (Ad5) has been more recently demonstrated to elicit much stronger Gag-specific CD8^+^ T-cell responses and protection from a Gag-expressing vaccinia virus in a mouse model^13^. Co-immunization with a plasmid DNA encoding SPD-gp100-CD40L, bearing the tumor gp100 antigen, and plasmids encoding IL-12p70 and granulocyte-macrophage colony stimulating factor (GM-CSF) has been shown to increase immune control of melanoma cells in mice^14^. However, the induction of MHC-II-restricted antigenic presentation or CD4^+^ T-cell initiation were not addressed in these studies. In contrast to these previous studies, our approach used polymers of M40 or S40 to generate, not only CD8^+^ T cells, but also, notably, (poly)functional CD4^+^ T-cell responses, without the need for additional adjuvant or immune-stimulatory molecules. This new property of LVs at inducing CD4^+^ T cells is of critical importance, as CD4^+^ T cells are critical immune players due to their: (i) long lifespan, (ii) direct effector functions, (iii) capacity to orchestrate the immune system by regulating innate immunity, (iv) helper functions in tailoring B-cell responses, and (v) helper functions in supporting CD8^+^ T-cell effector pathways^5^.

In this first study with this new-generation vector, we carried out the experiments using an integrative version of LV^24^. However, it should be noted that non-integrative LVs show similar immunogenicity if the injection dose is simply adjusted, as we have recently shown with the use of LVs in the development of a vaccine candidate against COVID-19^22,29,30^. Overall, we developed a new generation of LVs to target and activate DCs, route immunogens to the MHC-II pathway, and induce both CD4^+^ and CD8^+^ T-cell responses. Scaling up LV production for the preparation of large GMP batches has thus far been a limitation in the production of LV-based vaccines and the conducting of clinical trials. We have now transferred the technology to an industrial partner that circumvents this difficulty. In addition, the safety of LVs has been established in humans in a phase I/IIa human immunodeficiency virus-1 therapeutic vaccine trial^56^. The potential applications of this innovative strategy are much broader than anti-mycobacterial immunity and can be extended to LV-based vaccines against many other bacterial, viral, and parasitic infectious diseases and cancer.

## Materials and methods

### Ethical approval of animal studies

Experimentation on mice was realized in accordance with the European and French guidelines (Directive 86/609/CEE and Decree 87-848 of 19 October 1987) subsequent to approval by the Institut Pasteur Safety, Animal Care and Use Committee, protocol agreement delivered by local ethical committee # CETEA 2013–0036, # CETEA DAP180030, and CETEA 2012–0005 and Ministry of High Education and Research (APAFIS#14638-2018041214002048). The number of animals per experiment was discussed and decided with a bioinformatician, before approval of the protocols by the Institut Pasteur Safety, Animal Care and Use Committee, and Ministry of High Education and Research. A total number of 110 mice were used in this study. Immunization with LV and infection with Mtb in immunocompetent mice generate no pain, no suffering or distress. The study did not have humane endpoints.

### Construction of transfer pFLAP plasmids encoding for MBL or SPD collectin scaffolds, harboring selected mycobacterial antigens, CD40L and/or CCL20

Genes encoding for *Mus musculus* Mannan-Binding Lectin (MBL) or Surfactant-associated Protein D (SPD), engineered to harbor selected mycobacterial antigens and/or CCL20 within their collagen-like domains, and murine CD40L ectodomain instead of their CRD, were synthetized by GenScript after codon optimization. Each of these genes were inserted into the sites BamHI and XhoI of the transfer pFLAP∆U3 plasmid^57^. Transcription is under control of the native human CMV, human β2-microglobulin β2 m or BCUAG promoters, the two latter replacing CMV promoter after insertion between MluI and BamHI sites. The human β2-microglobulin promoter has been previously described^58^. The BCUAG promoter is a hybrid promoter comprising CMV enhancer, inflammation-related cis-regulating region and β2 m core promoter. The pFLAP∆U3 plasmid contains also a mutated WPRE (Woodchuck Posttranscriptional Regulatory Element) sequence to improve protein expression.

### Plasmid amplification and purification

Plasmid DNA were amplified in DH5α *Escherichia coli* in Lysogeny Broth (LB) completed with 50 μg/ml of kanamycin. The plasmid DNA was then purified by use of the NucleoBond Xtra Maxi EF Kit (Macherey Nagel). After drying, the DNA pellets were resuspended in Tris-EDTA Endotoxin-Free (TE-EF) buffer overnight, quantitated in a NanoDrop 2000c spectrophotometer (Thermo Scientific), adjusted to 1 μg/μl in TE-EF buffer, aliquoted and stored at −20 °C. The quality of the plasmid DNA was controlled: (i) either undigested or subsequent to digestion with a mixture of two plasmid-specific appropriate restriction enzymes prior to gel electrophoresis, and (ii) by sequencing the inserts in each pFLAP plasmid.

### Production and titration of LV

Non-replicative integrative LV were produced in Human Embryonic Kidney (HEK)293T cells, as previously detailed^57^. Briefly, 1 × 10^7^ cells/Petri dish were cultured in DMEM and were co-transfected in a tripartite manner with 1 ml of a mixture of: (i) 2.5 µg/ml of the pSD-GP-NDK packaging plasmid, encoding for codon optimized *gag-pol-tat-rre-rev*, (ii) 10 µg/ml of VSV-G Indiana envelop plasmid, and (iii) 10 µg/ml of “transfer” pFLAP plasmid in Hepes 1X containing 125 mM of Ca(ClO_3_)_2_. Supernatants were harvested at 48 h post-transfection, clarified by 6 min centrifugation at 2500 *rpm* and concentrated by 1 h ultracentrifugation at 22,000 *rpm* at 4 °C. LV were then aliquoted in PBS 1X, PIPES 20 mM, sucrose 2.5%, NaCl 75 mM and conserved at −80 °C.

To determine the titers of the produced LV, HEK293T were distributed at 4 × 10^4^ cell/well in flat-bottom 96-well-plates in complete DMEM in the presence of 8 µM aphidicolin (Sigma) to blocks the cell growth. The cells were then transduced with serial dilutions of concentrated LV. The titers, proportional to efficacy of the nuclear gene transfer, were determined as “Transduction Unit” (TU)/ml by quantitative real-time PCR on total lysates at day 3 post-transduction, by use of forward 5′-TGG AGG AGG AGA TAT GAG GG-3′ and reverse 5′-CTG CTG CAC TAT ACC AGA CA-3′ primers, specific to pFLAP plasmid and forward 5′-TCT CCT CTG ACT TCA ACA GC-3′ and reverse 5′-CCC TGC ACT TTT TAA GAG CC-3′ primers specific to the host housekeeping gene *gadph*, as described elsewhere^59^.

### Western blot

To generate an LV::S40-EsxH-StrepII, the pFlap-CMV-GFP-WPREm plasmid was digested with BamHI and XhoI. Fragments encoding S40-H or StrepII were amplified by PCR. These three fragments were purified on 1% agarose gel using the NucleoSpin Gel and PCR Clean-up kit (Macherey Nagel). Following the recommendations of the In-Fusion^®^ HD Cloning Plus kit (Takara), assembling of the fragments encoding for S40-H or -StrepII with the plasmid was carried out using the Premix Enzyme and 15 min incubation at 50 °C. The assembled plasmid was used to transform *E. coli* (Stellar Competent Cells) and the transformants have been selected on kanamycin. The full sequence of the insert was checked by sequencing. Integrative LV::S40-EsxH-StrepII was produced as described above. HEK293T cells were transduced with MOI of 100 of LV::S40-EsxH-StrepII and supernatants and cell lysates were prepared 96 h later. Samples (20 µg/assay) were analyzed as such or were treated with reducing agent (XT reagent containing β-Mercaptoethanol, Biorad) and heated at 95 °C for 5 min. Samples were loaded on a NuPAGE Bis-Tris 4–12% gradient dodecylsulfate-polyacrylamide gel (Thermo). After 2H of migration at 30 mA, the separated proteins were transferred to PVDF membrane by 16 h electro-transfer at 30 V in a NuPAGE transfer system (Thermo) following the manufacturer’s recommendations. Membranes were then blocked with fat-free bovine milk and incubated for 2 h with mouse anti-StrepII Tag C23.21 mAb, diluted at 1/1000 in Tris buffered saline containing 0.5% Tween_20_ and 5% fat-free bovine milk. Horseradish peroxidase-conjugated anti-mouse IgG (Cell signaling) was used at 1/40,000 for detection, followed by incubation with Enhanced ChemiLuminescence substrate (Super signal West PICO plus, Thermo). Blots were imaged in a Chemidoc XRS + system and analyzed with Image Lab software (Biorad).

### Exclusion chromatography

Supernatants were generated by transducing 5 × 10^5^ HEK293T cells with LV::S40-H-StrepII (MOI = 100). After 5 h, medium was replaced with Freestyle 293 Expression Medium (Gibco). After 48 h, supernatant was harvested, clarified on a 0.2 µm pore size filter (Sartorius), and 50× concentrated using an Ultra-15 Centrifugal Filter Units (Amicon). Fast protein liquid chromatography (ÄKTA pure protein purification system, Cytivia) using a Superose 6 HR 10/30 gel filtration column was used for further sizing the protein multimerization. The column was equilibrated with 50 mM Phosphate Buffer (pH 7.5), 150 mM NaCl at 0.5 ml/min for 3 h. The concentrated proteins (0,1 ml) were injected on the column with a 0,1 ml loop and proteins were eluted at a flow rate of 0.5 ml/ml using the same buffer. Fractions (0.5 ml) were collected in a 96 deep well plate. The column was first calibrated with “standard” proteins of known size and weight, with Gel Filtration Calibration Kits (Cytivia HMW) under the same conditions. The column void volume was determined with Blue Dextran 2000. Elution fraction were analyzed by Western blot in denaturing condition as described above.

### Mice, immunization

Female C57BL/6JRj or BALB/cJ mice (Janvier, Le Genest Saint Isle, France) were used between the age of 7 and 10 weeks. Seven mice were housed per cage. Mice were immunized subcutaneously (s.c.) at the basis of the tail with the indicated amounts of LV contained in 200 µl. When indicated, mice were immunized intranasally (i.n.) with the indicated amounts of LV contained in 20 µl, as previously detailed^28^. The i.n. administration was realized under anesthesia, obtained by peritoneal injection of a mixture of Xylazine (Rompun, 10 mg/kg) and Ketamine (Imalgene, 100 mg/kg).

### T-cell assay by ELISPOT

At day 11–14 post-immunization, splenocytes from individual mice (*n* = 3/group) were homogenized and filtered through 100 μm-pore filters and centrifuged at 1300 *rpm* during 5 min. Cells were then treated with Red Blood Cell Lysing Buffer (Sigma), washed twice in PBS and counted in a MACSQuant10 cytometric system (Miltenyi Biotec). Splenocytes were then seeded at 0.5–1 × 10^5^ cells /well in 200 µl of RPMI-GlutaMAX, containing 10% heat-inactivated FBS, 100 U/ml penicillin and 100 µg/ml streptomycin, 1 × 10^−4 ^M non-essential amino-acids, 1% vol/vol HEPES, 1 × 10^−3 ^M sodium pyruvate and 5 × 10^−5 ^M of β-mercapto-ethanol in the wells of IFN-y or TNF-α ELISPOT plates (Mouse ELISPOT^PLUS^, Mabtech). Cells were left unstimulated or were stimulated with 2 µg/ml of synthetic peptide (Proteogenix, Strasbourg, France), harboring the well-defined MHC-I-, or -II-restricted T-cell epitopes of each mycobacterial antigen. In parallel, splenocytes were stimulated with 2.5 µg/ml of Concanavalin A (Sigma), as a functionality control. For each individual, the assays were run in technical triplicates, following Mabtech’s recommendations. Spots were quantified in an ELR04 ELISPOT reader (AID, Strassberg, Germany).

### T-cell assay by intracellular cytokine staining, lung T-cell phenotyping

Splenocytes from immunized mice were obtained by tissue homogenization and passage through 100 μm-pore filter and were cultured during 6 h at 8 × 10^6^ cells/well in 24-well plates in the presence of 10 μg/ml of homologous or control peptide, 1 μg/ml of anti-CD28 (clone 37.51) and 1 μg/ml of anti-CD49d (clone 9C10-MFR4.B) mAbs (BD Pharmingen). During the last 3 h of incubation, cells were added with a mixture of Golgi Plug and Golgi Stop (BD Pharmingen). Cells were then collected, washed with PBS containing 3% heat-inactivated FBS and 0.1% NaN_3_ (FACS buffer) and incubated for 25 min at 4 °C with a mixture of FcγII/III receptor blocking anti-CD16/CD32 (clone 2.4G2), APC eF780-anti-CD3ε (clone 17A2), eFluor450-anti-CD4 (RM4-5) and BV711-anti-CD8α (53-6.7), mAbs (BD Pharmingen and eBioscience). Cells were then washed twice in FACS buffer, permeabilized by use of Cytofix/Cytoperm kit (BD Pharmingen). Cells were then washed twice with PermWash 1x buffer from the Cytofix/Cytoperm kit and incubated with a mixture of FITC-anti-IL-2 (clone JES6-5H4, eBioscience), PE-Dazzle-anti-TNF-α (MP6-XT22, Biolegend) and APC-anti-IFN-γ (clone XMG1.2, BD Pharmingen) mAbs or a mixture of appropriate control Ig isotypes, during 30 min at 4 °C. Cells were then washed twice in PermWash and once in FACS buffer and then fixed with Cytofix (BD Pharmingen) overnight at 4 °C. The cells were acquired in an Attune NxT cytometer system (Invitrogen). Data were analyzed by FlowJo software (Treestar, OR, USA). Lung T-cell phenotyping was performed as recently described^25^.

### Antigenic presentation assay

Bone-marrow derived DC were plated at 5 × 10^5^ cells/well in 24-well plates in RPMI 1640 containing 5% FBS. Cells were transduced with LV or were loaded homologous or control synthetic peptides. At 24 h post infection 5 × 10^5^ appropriate T-cell hybridomas^60^ were added and the culture supernatants were quantitated for IL-2 production at 24 h by ELISA. Synthetic peptides were synthesized by Proteogenix (Schiltigheim, France).

### In vitro migration assay

Supernatants were generated by transducing 5 × 10^5^ HEK293T cells with LV::S40-HAPEHR or LV::S40-HAPEHR-20 (MOI = 100) under control of BCUAG promoter. After 5 h, medium was replaced with RPMI 1640 containing 100 U/ml penicillin and 100 µg/ml streptomycin. After 48 h, supernatant was harvested and clarified on a 0.2 µm pore size filter (Sartorius). Untransduced HEK293T cell supernatant was use as a negative control. Same supernatant complemented with 400 ng/ml of recombinant mouse CCL20 protein (R&D systems) was used as a positive control. Transwell migration assays were performed using 8 μm pore size cell culture inserts (BD Falcon, Franklin Lakes, NJ) in 24-well culture plates (BD Falcon). Bone-marrow derived DCs were plated at 5 × 10^5^ cells/well in the upper chamber in 100 μl RPMI 1640 containing 1% bovine serum albumin. Bottom chamber was filled with 600 μl of supernatant. After 4 h at 37 °C, cells in the bottom chamber were counted using a Nucleocounter NC-200 (Chemometec).

### Protection assay

Mtb H37Rv strain, Danish BCG 1331 SSI strain or BCG::ESX-1^Mmar^ ^27^, were cultured in Dubos broth, complemented with Albumine, Dextrose and Catalase (ADC, Difco, Becton Dickinson, Le Pont-de-Claix, France). Experiments with pathogenic mycobacteria were performed in BSL3, following the hygiene and security recommendations of Institut Pasteur. Five mice were housed per cage. C57BL/6 mice were primed s.c. with 1 × 10^6^ CFU/mouse of Danish BCG or BCG::ESX-1^Mmar^ ^27^, at day 0, boosted s.c. with 5 × 10^8^ TU of SPD40-HAPEHR-20 at week 5, and boosted i.n. with 5 × 10^8^ TU of SPD40-HAPEHR-20 at week 10. The mice were challenged 2 weeks after the mucosal boost by use of a homemade nebulizer via aerosol, as previously described^28^. Briefly, 5 ml of a suspension of 1.7 × 10^6^ CFU/ml of Mtb H37Rv strain were aerosolized in order to deliver an inhaled dose of ≈200 CFU/mouse. Alternatively, the Mtb challenge was performed via nasal route by 10^3^ CFU/mouse. The challenged mice were then placed in isolator. Five weeks later, lungs or spleen of the infected mice were homogenized by using a MillMixer homogenizer (Qiagen, Courtaboeuf, France) and serial fivefold dilutions prepared in PBS were plated on 7H11 Agar complemented with ADC (Difco, Becton Dickinson). CFU were counted after 3 weeks of incubation at 37 °C. Statistical significance of inter-group Mtb load differences was determined by Mann–Whitney *t*-test by use of Prism v8.01 (GraphPad Software, Inc.).

### Lung histopathology

Histopathological analysis was performed on the left lung lobes, fixed in formalin and embedded in paraffin. Five µm thick sections were stained with Hematoxylin and Eosin (H&E). Granulomatous and inflammatory lesions were qualitatively described and granuloma sizes were measured on images acquired on an Axioscan Z1 Zeiss slide scanner, using the Zen 2 blue edition software.

### Statistical information

Significance of differences between experimental groups was evaluated by using Mann–Whitney test, as mentioned in each figure legend. Statistical analyses were performed using GraphPad Prism v8.01 (GraphPad Software, CA, USA).

Data from the study are available upon request from the corresponding author.

## Supplementary information

Supplementary Information

Supplementary Checklist
