# Supplementary material for: A lentiviral vector expressing a dendritic cell-targeting multimer induces mucosal anti-mycobacterial CD4+ T-cell immunity
Source: Mucosal Immunol. 2022 Sep 14;15(6):1389–404. doi: 10.1038/s41385-022-00566-z (PMC9473479; doi:10.1038/s41385-022-00566-z)
Supplement: Supplementary file 1 — Supplementary Information [file 41385_2022_566_MOESM1_ESM.pdf]

Supplementary Information for

A Lentiviral Vector Expressing a Dendritic Cell-Targeting Multimer Induces Mucosal anti-Mycobacterial $CD4^+$ T-Cell Immunity

François Anna[1*], Jodie Lopez[1], Fanny Moncoq[1], Catherine Blanc[1], Pierre Authié[1], Amandine Noirat[1], Ingrid Fert[1], Philippe Souque[1], Fabien Nevo[1], Alexandre Pawlik[2], David Hardy[3], Sophie Goyard[4], Denis Hudrisier[5], Roland Brosch[2], Françoise Guinet[6], Olivier Neyrolles[5], Pierre Charneau[1], and Laleh Majlessi[1*]

*Corresponding author. Email: francois.anna@pasteur.fr

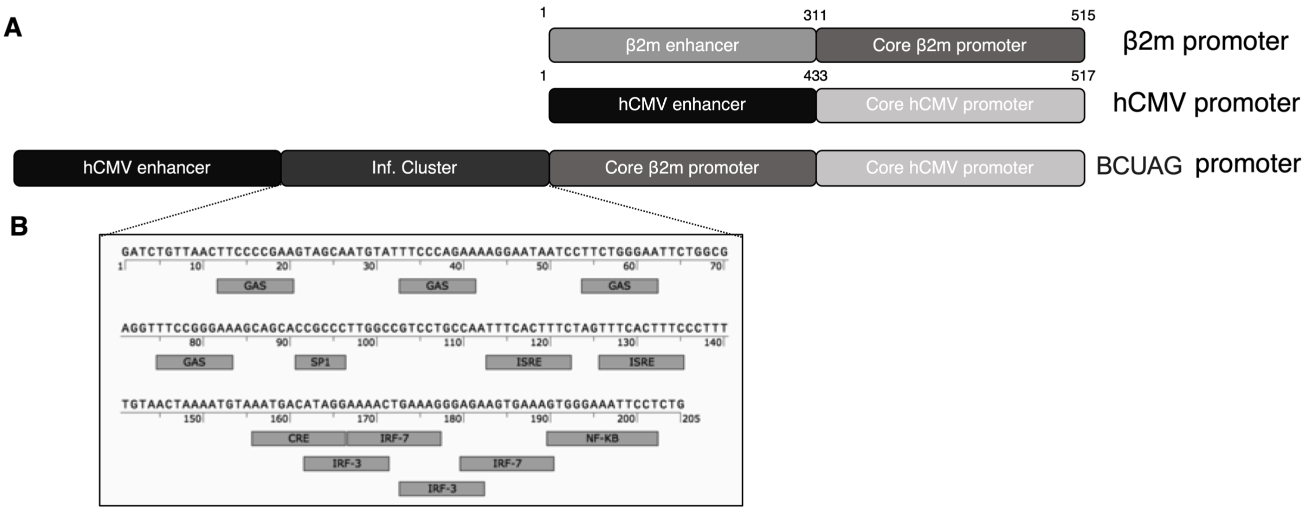

Fig. S1. (A) Schematic description of the three used promoters. β2m promoter (Seq: LRG-1215; from 4556 to 5070), hCMV promoter (RefSeq: MN920393.1; from 174188 to 174714), BCUAG is a combination of β2m and hCMV promoters with the addition of "Inf" (Inflammation-related) cluster, a set of cis-regulating motifs associated with inflammation. **(B)** Sequence of Inf Cluster. Cis regulating motifs and associated transcriptional factor are indicated under the sequence.

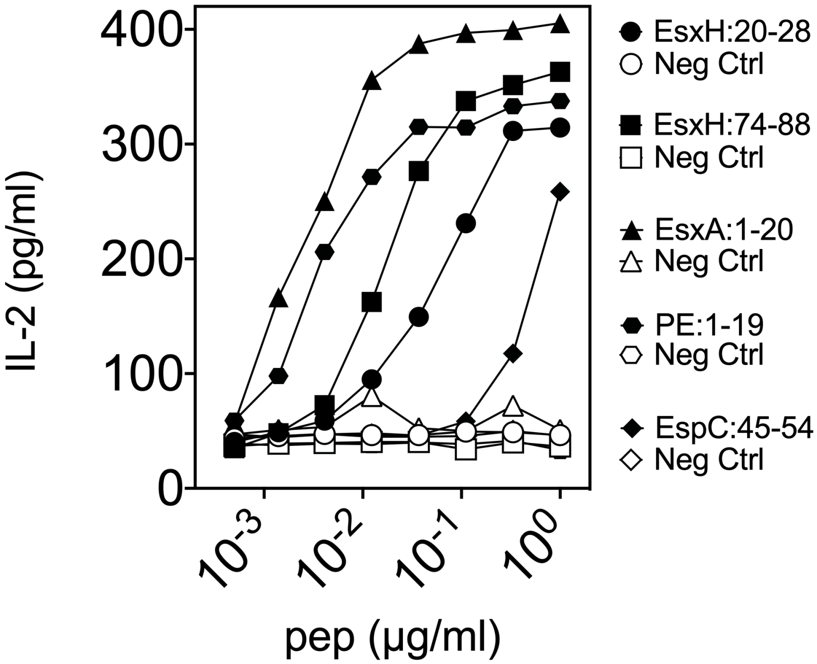

Fig. S2. Sensitivity of the T-cell hybridomas used in the Mtb antigen presentation assays. BM-DC from BALB/c (H-2^d) or C57BL/6 (H-2^b) mice were incubated with various concentrations of homologous or negative control peptides. At day 1, presentation of MHC-I- or -II-restricted epitopes were assessed by use of T-cell hybridomas specific to EsxH:20-28 (YB8, restricted by K^d), EsxH:74-88 (1G1, restricted by I-A^d), EsxA:1-20 (NB11, restricted by I-A^b), PE:19:1-18 (IF6, restricted by I-A^b), or EspC:45:54 (IF1, restricted by I-A^b). Results are concentrations of IL-2 produced by T-cell hybridomas 24h after the T-cell addition.

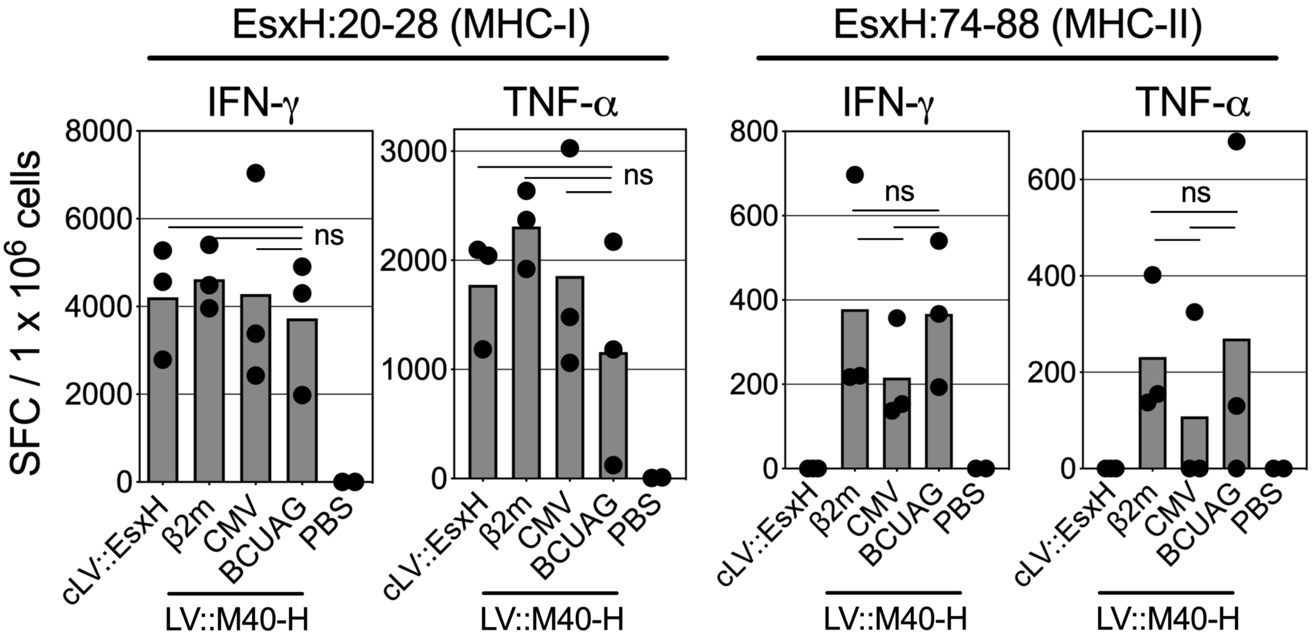

Fig. S3. T-cell immunogenicity of LV encoding for M40-H evaluated by ELISPOT. IFN-γ- or TNF-α-producing $CD8^+$ (left) or $CD4^+$ (right) T-cell responses, as assessed by ELISPOT at day 13 post-immunization, in the spleen of individual BALB/c mice (*n* = 3), immunized s.c. with 1×10^8 TU/mouse of a conventional LV::EsxH harboring β2m promoter or LV::M40-H, harboring β2m, CMV or BCUAG promoters. Frequencies of Spot Forming Cells (SFC) were determined subsequent to in vitro stimulation of splenocytes with EsxH:20-28 (left) or EsxH:74-88 (right) synthetic peptide. Quantitative differences between various groups were evaluated by non-parametric Mann & Whitney test, $p < 0.05$. ns = non-significant.

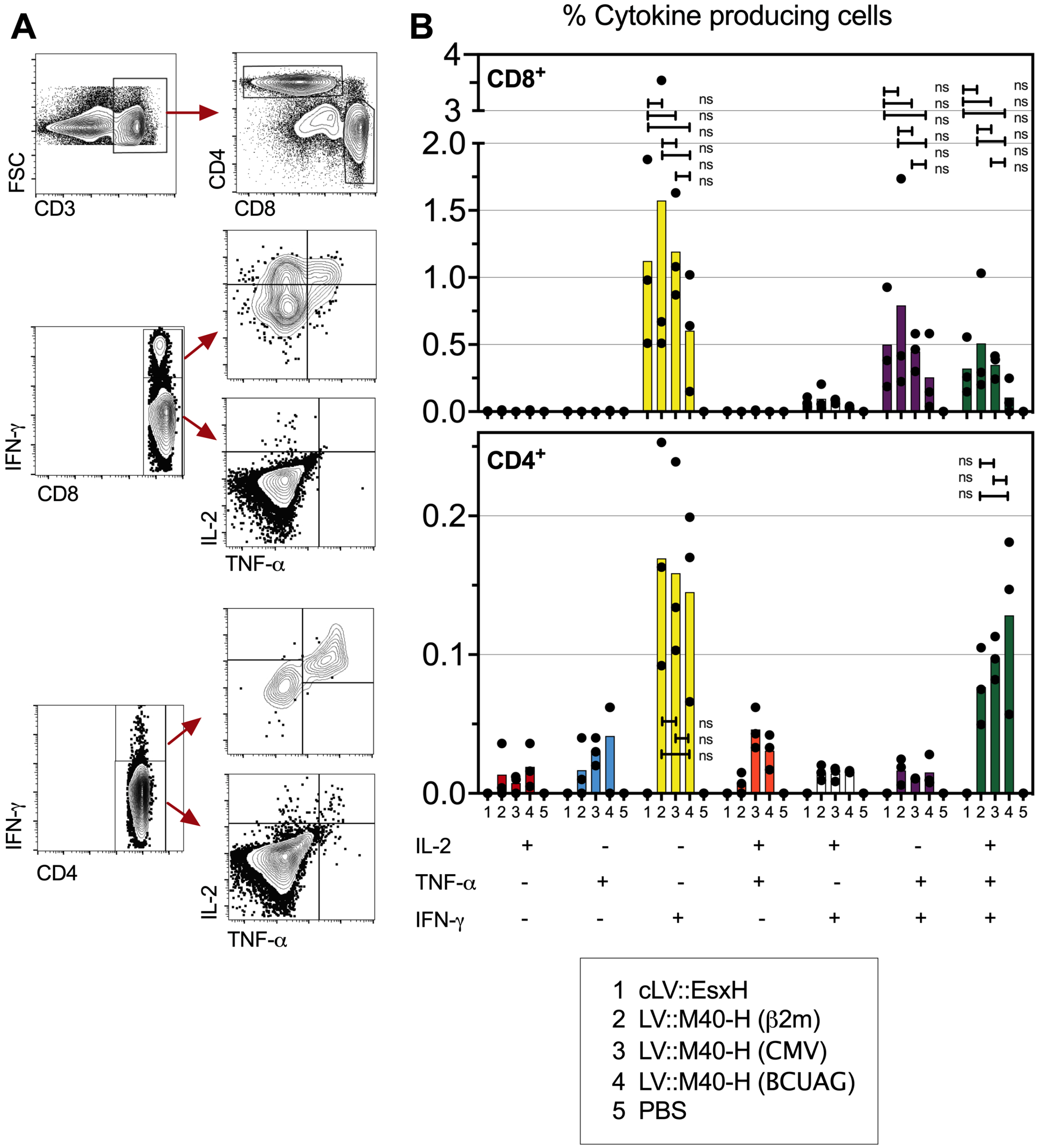

Fig. S4. T-cell immunogenicity of LV encoding for M40-H evaluated by ICS. BALB/c mice ($n = 3$), immunized s.c. with 1×10^8 TU/mouse of a conventional LV::EsxH harboring β2m promoter or LV::M40-H, harboring β2m, CMV or BCUAG promoters. At day 13 post-immunization, ICS analyses were performed in the spleen of individual mice. **(A)** Cytometric gating strategy of $CD4^+$ or $CD8^+$ T splenocytes and representative IFN-γ^+ or IFN-γ^- $CD8^+$ or $CD4^+$ T cells, expressing TNF-α and/or IL-2. **(B)** Recapitulative percentages of each functional subsets within the $CD8^+$ (top) or $CD4^+$ (bottom) T-cell population. Quantitative differences between various groups for each functional T-cell population were not statistically significant (non-parametric Mann & Whitney test, $p < 0.05$).

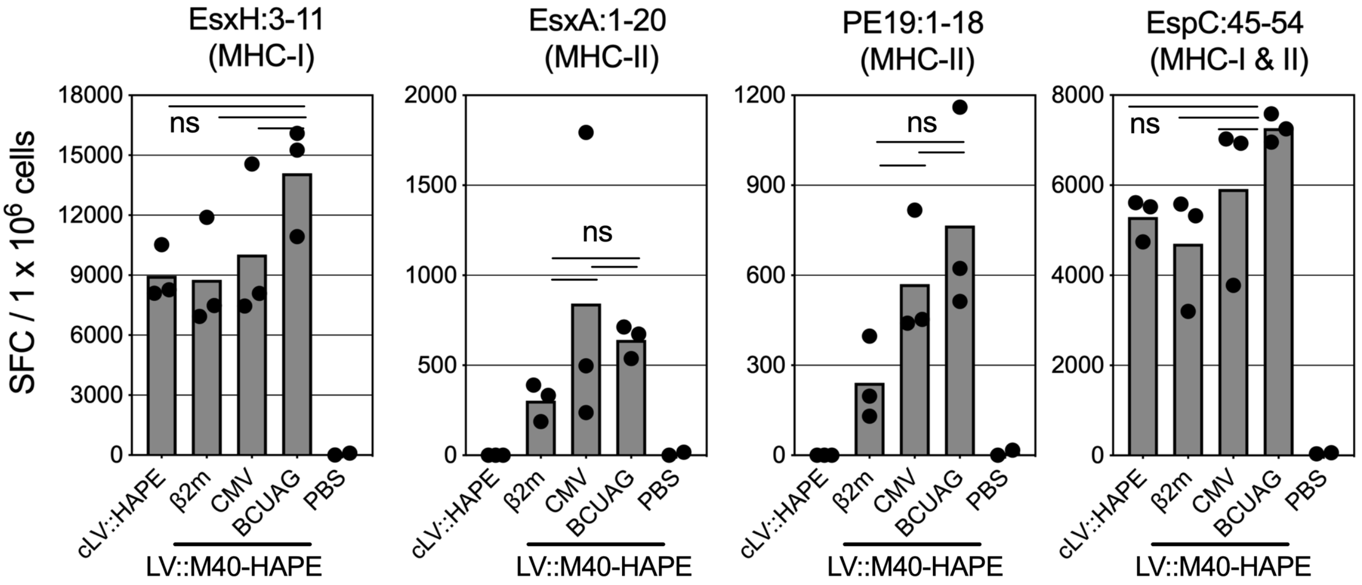

Fig. S5. Immunogenicity of the poly-antigenic LV::M40-HAPE evaluated by ELISPOT. (A) IFN-γ T-cell responses, as assessed by ELISPOT at day 14 post-immunization, in the spleen of individual C57BL/6 mice (n = 3), immunized s.c. with 1×10^8 TU/mouse of a conventional LV::HAPE harboring β2m promoter or LV::M40-HAPE harboring β2m, CMV or BCUAG promoters. The frequencies of responding T cells were determined subsequent to in vitro stimulation with EsxH:3-11 (containing MHC-I-restricted epitope) or EsxA:1-20 (containing MHC-II-restricted epitope), PE10:-1-18 (containing MHC-II-restricted epitope), or EspC:45-54 (containing both MHC-I and II-restricted epitopes) synthetic peptide. Quantitative differences between various groups were evaluated by non-parametric Mann & Whitney test, $p < 0.05$. ns = non-significant.

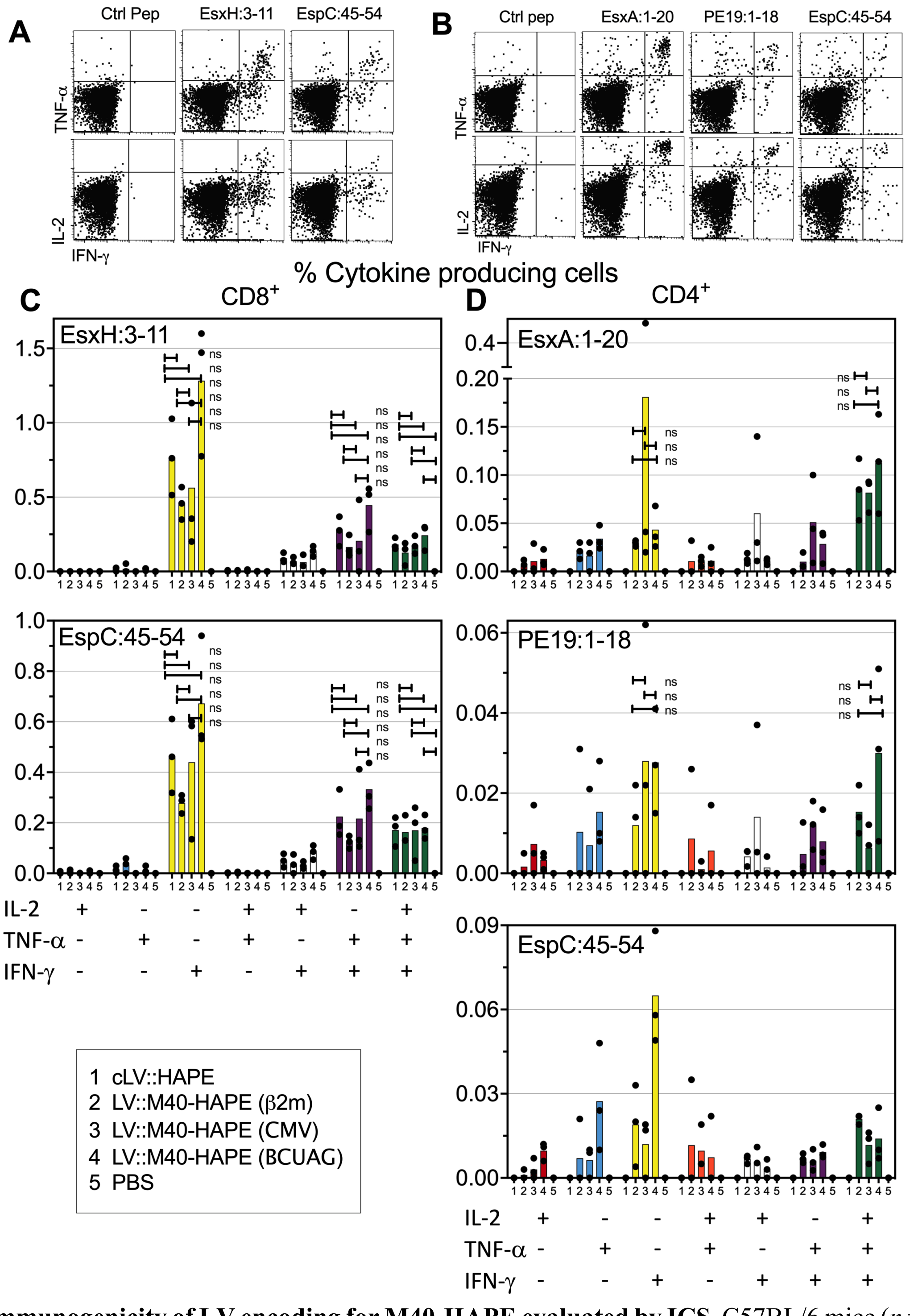

Fig. S6. T-cell immunogenicity of LV encoding for M40-HAPE evaluated by ICS. C57BL/6 mice ($n = 3$), immunized s.c. with 1×10^8 TU/mouse of a conventional LV::HAPE harboring β2m promoter or LV::M40-HAPE, harboring β2m, CMV or BCUAG promoters. At day 14 post-immunization, ICS analyses were performed in the spleen of individual mice. **(A)** Representative cytometric dot blots showing IFN-γ^+ or IFN-γ^- CD8$^+$ or CD4$^+$ T cells, expressing TNF-α and/or IL-2. **(C)** Recapitulative percentages of each functional subsets within the CD8$^+$ (left) or CD4$^+$ (right) T-cell population, subsequent to in vitro stimulation with EsxH:3-11 (containing MHC-I-restricted epitope) or EsxA:1-20 (containing MHC-II-restricted epitope), PE10:-1-18 (containing MHC-II-restricted epitope), or EspC:45-54 (containing MHC-I and II-restricted epitopes) synthetic peptide. Quantitative differences between various groups for each functional T-cell population were not statistically significant (non-parametric Mann & Whitney test, $p < 0.05$).

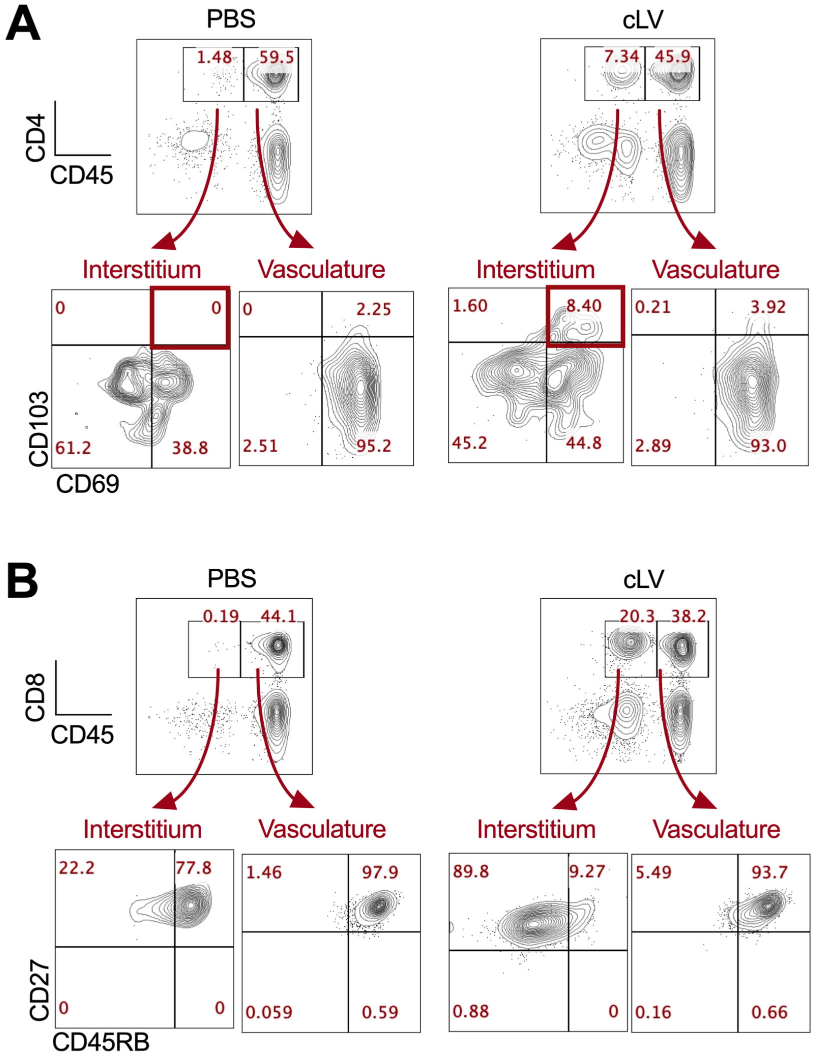

Fig. S7. Features of mucosal CD4$^+$ or CD8$^+$ T cells in mice immunized i.n. with a conventional LV::HAPE. Shown are lung CD4$^+$ **(A)** or CD8$^+$ **(B)** T cells, distinguished for their location within the interstitium (CD45$_{i.v}^-$) or in the vasculature (CD45$_{i.v}^+$). **(A)** Profile of CD103 vs CD69 of the lung CD4$^+$ T cells. **(B)** Profile of CD27 vs CD45RB of the lung CD8$^+$ T cells.

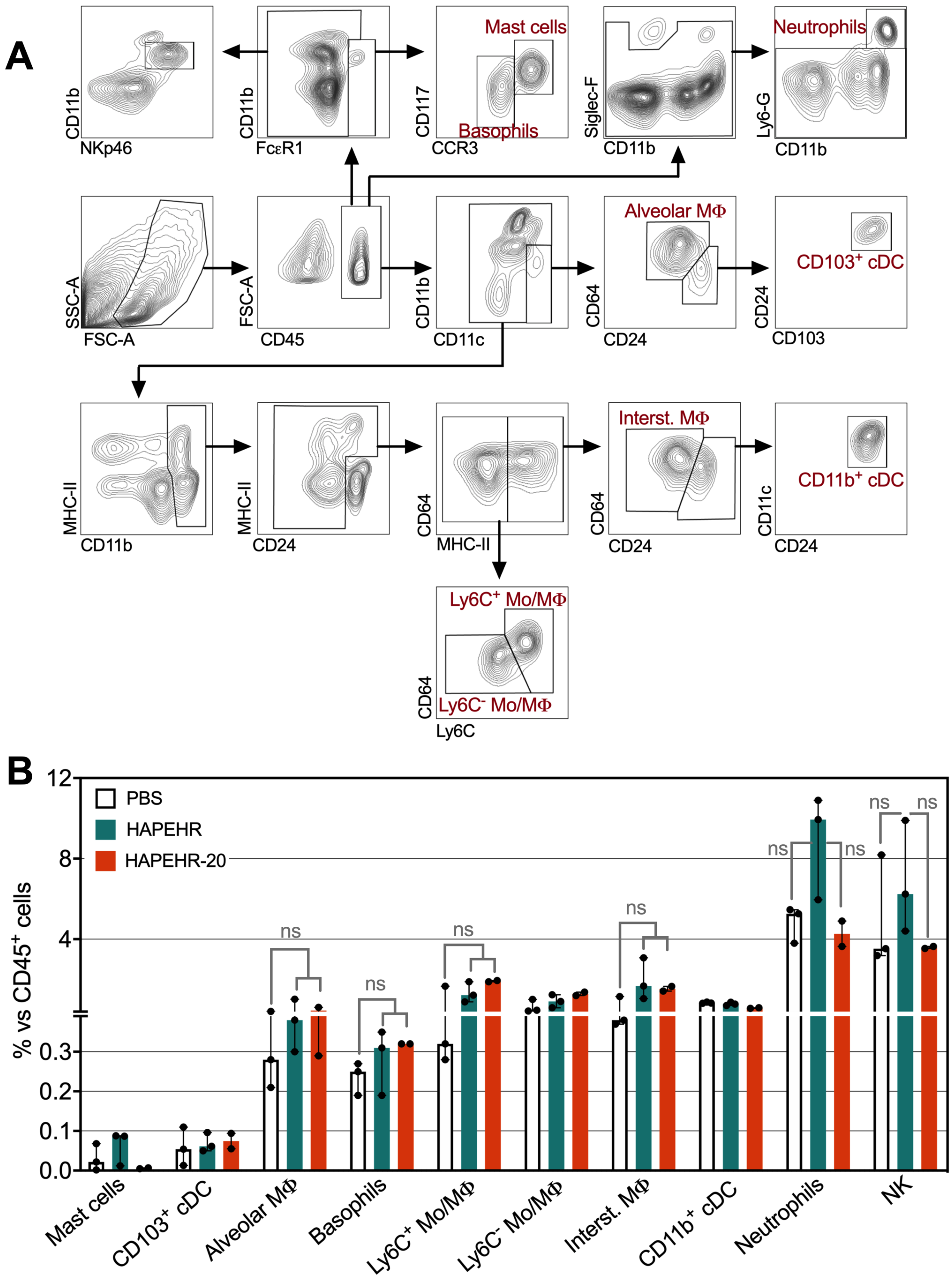

Fig. S8. Features of lung innate immune cells after i.n. administration of LV::S40 HAPEHR or LV::S40 HAPEHR-20. Flow cytometric gating approach to identify and quantify various lung innate immune cell subsets. Hematopoietic $CD45^+$ cells were analyzed by use of antibodies specific to combination of surface markers, allowing identification of innate immune cell subsets. **(B)** Percentages of each innate immune subset versus total lung $CD45^+$ cells at day 1 after injection of PBS, LV::S40 HAPEHR or LV::S40 HAPEHR-20. The weak differences observed were statistically insignificant. Statistical significance was evaluated by Mann-Whitney test, $p < 0.05$, ns = non-significant.

Table S1. Mtb proteins rationally selected as target antigens to be incorporated in the prospective multistage anti-TB LV.

**Mtb immunogen**	**Locus in H37Rv**	**Size a.a**	**Major characteristics**
EsxA	*rv3875*	95	Early Secreted Antigenic Target 6 kDa (ESAT-6) secreted by ESX-1 T7SS
EspC	*rv3615c*	103	ESX-1 secretion-associated proteins C secreted by ESX-1 T7SS
EsxH	*rv0288*	96	Virulence-related factor (TB10.4) secreted by ESX-3 T7SS
PE19	*rv1791*	99	Virulence-related factor, with numerous homologous secreted by ESX-5 T7SS
Hrp1	*rv2626c*	143	Dormancy-related Hypoxic response protein 1
RpfD	*rv2389c*	154 (42-154)*	Reactivation-related Resuscitation promoting factor D (mb)

*Only the $RpfD_{42-154}$ ectodomain was included to minimize the hydrophobicity of the resulted protein.

Table S2. Various M40 and S40 scaffolds designed to harbor the selected Mtb antigens and/or CCL20.

Carrier	Antigens	Chemo-attractant	Nomenclature	Monomer Lenght (a.a)
MBL40	EsxH	—	M40-H	414
	EsxH-EsxA	—	M40-HA	556
	EsxH-EsxA-PE19	—	M40-HAP	721
	EsxH-EsxA-PE19-EspC	—	M40-HAPE	885
SPD40	EsxH	—	S40-H	506
	EsxH-EsxA-PE19-EspC	—	S40-HAPE	736
	EsxH-EsxA-PE19-EspC-Hrp1-RpfD	—	S40-HAPEHR	1004
	EsxH-EsxA-PE19-EspC-Hrp1-RpfD	CCL20	S40-HAPEHR-20	1128

Table S3. Sequences of MBL fused with selected Mtb antigens and CCL20 as coded by LV.

Poly-antigenic LV	Insert length (a.a.)	Sequence
LV::M40-EsxH<br><br>(LV::M40-H)	414	MSIFTSFLLLCVVTVVYAETLTEGVQNSCPVVTCSSPGLNGFPGKDGRDGAKGEKGEPGQ<br>GLRGLQGPPGAVGPTGPPGNPGLKGAVGPKGDRGDRGGGSQIMYNYPAMLGHAGDMA<br>GYAGTLQSLGAEIAVEQAALQSAWQGDTGITYQAWQAQWNQAMEDLVRAYHAMSSTH<br>EANTMAMMARDTAEAAKWGGGSGLRGLQGPPGALGPPGSVGSPGSPGPKGQKGDHGD<br>NRAIEEKLANMEAEIRILKSKLQLTNKLHAFSMGGGSGDEDPQIAAHVVSEANSNAASVL<br>QWAKKGYYTMKSNLVMLENGKQLTVKREGLYYVYTQVTFCSNREPSSQRPFIVGLWLK<br>PSSGSERILLKAANTHSSSQLCEQQSVHLGGVFELQAGASVFVNVTEASQVIHRVGFSSFG<br>LLKL
LV::M40-EsxH-EsxA<br><br>(LV::M40-HA)	556	MSIFTSFLLLCVVTVVYAETLTEGVQNSCPVVTCSSPGLNGFPGKDGRDGAKGEKGEPGQ<br>GLRGLQGPPGAVGPTGPPGNPGLKGAVGPKGDRGDRGGGSQIMYNYPAMLGHAGDMA<br>GYAGTLQSLGAEIAVEQAALQSAWQGDTGITYQAWQAQWNQAMEDLVRAYHAMSSTH<br>EANTMAMMARDTAEAAKWGGGSGFPGPPGPKGEPGSPAGRGERGFQGSPGKMGPAGS<br>KGEPGGGSGTEQQWNFAGIEAAASAIQGNVTSIHSLLDEGKQSLTKLAAAWGGSGSEAY<br>QGVQQKWDATATELNNALQNLARTISEAGQAMASTEGNVTGMFAGGGSGLRGLQGPP<br>GALGPPGSVGSPGSPGPKGQKGDHGDNRAIEEKLANMEAEIRILKSKLQLTNKLHAFSMG<br>GGSGDEDPQIAAHVVSEANSNAASVLQWAKKGYYTMKSNLVMLENGKQLTVKREGLY<br>YVYTQVTFCSNREPSSQRPFIVGLWLKPSSGSERILLKAANTHSSSQLCEQQSVHLGGVFE<br>LQAGASVFVNVTEASQVIHRVGFSSFGLLKL
LV::M40-EsxH-EsxA-PE19<br><br>(LV::M40-HAP)	721	MSIFTSFLLLCVVTVVYAETLTEGVQNSCPVVTCSSPGLNGFPGKDGRDGAKGEKGEPGQ<br>GLRGLQGPPGAVGPTGPPGNPGLKGAVGPKGDRGDRGGGSQIMYNYPAMLGHAGDMA<br>GYAGTLQSLGAEIAVEQAALQSAWQGDTGITYQAWQAQWNQAMEDLVRAYHAMSSTH<br>EANTMAMMARDTAEAAKWGGGSGFPGPPGPKGEPGSPAGRGERGFQGSPGKMGPAGS<br>KGEPGGGSGTEQQWNFAGIEAAASAIQGNVTSIHSLLDEGKQSLTKLAAAWGGSGSEAY<br>QGVQQKWDATATELNNALQNLARTISEAGQAMASTEGNVTGMFAGGGSGLPGRDGRD<br>GREGPRGEKGDPGLPGPMGLSGLQGPTGPVGPKGENGSAGEPGPKGERGLSGGGGSFVT<br>TQPEALAAAAANLQGIGTTMNAQNAAAAAPTTGVVPAAADEVSALTAAQFAAHAQMY<br>QTVSAQAAAIHEMFVNTLVASSGSYAATEAANAAAAGGGSGLRGLQGPPGALGPPGSV<br>GSPGSPGPKGQKGDHGDNRAIEEKLANMEAEIRILKSKLQLTNKLHAFSMGGGSGDEDP<br>QIAAHVVSEANSNAASVLQWAKKGYYTMKSNLVMLENGKQLTVKREGLYYVYTQVTF<br>CSNREPSSQRPFIVGLWLKPSSGSERILLKAANTHSSSQLCEQQSVHLGGVFELQAGASVF<br>VNVTEASQVIHRVGFSSFGLLKL
LV::M40-EsxH-EsxA-PE19-EspC<br><br>(LV::M40-HAPE)	885	MSIFTSFLLLCVVTVVYAETLTEGVQNSCPVVTCSSPGLNGFPGKDGRDGAKGEKGEPGQ<br>GLRGLQGPPGAVGPTGPPGNPGLKGAVGPKGDRGDRGGGSQIMYNYPAMLGHAGDMA<br>GYAGTLQSLGAEIAVEQAALQSAWQGDTGITYQAWQAQWNQAMEDLVRAYHAMSSTH<br>EANTMAMMARDTAEAAKWGGGSGFPGPPGPKGEPGSPAGRGERGFQGSPGKMGPAGS<br>KGEPGGGSGTEQQWNFAGIEAAASAIQGNVTSIHSLLDEGKQSLTKLAAAWGGSGSEAY<br>QGVQQKWDATATELNNALQNLARTISEAGQAMASTEGNVTGMFAGGGSGLPGRDGRD<br>GREGPRGEKGDPGLPGPMGLSGLQGPTGPVGPKGENGSAGEPGPKGERGLSGGGGSFVT<br>TQPEALAAAAANLQGIGTTMNAQNAAAAAPTTGVVPAAADEVSALTAAQFAAHAQMY<br>QTVSAQAAAIHEMFVNTLVASSGSYAATEAANAAAAGGCPGLPGAAGPKGEAGAKGDR<br>GESGLPGIPGKEGPTGPKGNQGEKGIRGEKGDSGPSGGGSTENLTVQPERLGVLASHHDN<br>AAVDASSGVEAAAGLGESVAITHGPYCSQFNDTLNVYLTAHNALGSSLHTAGVDLAKSL<br>RIAAKIYSEADEAWRKAIDGLFTGGGSGLRGLQGPPGALGPPGSVGSPGSPGPKGQKGDH<br>GDNRAIEEKLANMEAEIRILKSKLQLTNKLHAFSMGGGSGDEDPQIAAHVVSEANSNAAS<br>VLQWAKKGYYTMKSNLVMLENGKQLTVKREGLYYVYTQVTFCSNREPSSQRPFIVGLW<br>LKPSSGSERILLKAANTHSSSQLCEQQSVHLGGVFELQAGASVFVNVTEASQVIHRVGFSS<br>FGLLKL

Complete sequences of EsxH, EsxA, EspC and PE19, with or without CCL20.

Table S4. Sequences of SPD fused with selected Mtb antigens and CCL20 as coded by LV.

Polyantigenic LV	Insert length (a.a.)	Sequence
LV::S40-EsxH<br><br>(LV::S40-H)	506	MLPFLSMLVLLVQPLGNLGAEMKSLSQRSVPNTCTLVMCSPTENGLPGRDGRDGREGPRGEKGDPGLPG<br>PMGLSGLQGPTGPVGPKGENGSAGEPGPKGERGLSGGSGSQIMYNYPAMLGHAGDMAGYAGTLQSLGA<br>EIAVEQAALQSAWQGDTGITYQAWQAQWNQAMEDLVRAYHAMSSTHEANTMAMMARDTAEAAKWG<br>GGSGPPGLPGIPGPAGKEGPSGKQGNIGPQGKPGPKGEAGPKGEVGAPGMQGSTGAKGSTGPKGERGAPG<br>VQGAPGNAGAAGPAGPAGPQGAPGSRGPPGLKGDRGVPGDRGIKGESGLPDSAALRQQMEALKGKLQR<br>LEVAFSHYQKAALFPDGGGSGDEDPQIAAHVVSEANSNAASVLQWAKKGYYTMKSNLVMLENGKQLTV<br>KREGLYYVYTQVTFCSNREPSSQRPFIVGLWLKPSSGSERILLKAANTHSSSQLCEQQSVHLGGVFELQAG<br>ASVFVNVTEASQVIHRVGFSSFGLLKL
LV::S40-EsxH-EsxA-PE19-EspC<br><br>(LV::S40-HAPE)	736	MLPFLSMLVLLVQPLGNLGAEMKSLSQRSVPNTCTLVMCSPTENGLPGRDGRDGREGPRGEKGDPGLPG<br>PMGLSGLQGPTGPVGPKGENGSAGEPGPKGERGLSGGSGSQIMYNYPAMLGHAGDMAGYAGTLQSLGA<br>EIAVEQAALQSAWQGDTGITYQAWQAQWNQAMEDLVRAYHAMSSTHEANTMAMMARDTAEAAKWG<br>GGSGGTEQQWNFAGIEAAASAIQGNVTSIHSLLDEGKQSLTKLAAAWGGSGSEAYQGVQQKWDATATE<br>LNNALQNLARTISEAGQAMASTEGNVTGMFAGGGSGGSFVTTQPEALAAAAANLQGIGTTMNAQNAAA<br>AAPTTGVVPAAADEVSALTAAQFAAHAQMYQTVSAQAAAIHEMFVNTLVASSGSYAATEAANAAAAG<br>GGSGGTENLTVQPERLGVLASHHDNAAVDASSGVEAAAGLGESVAITHGPYCSQFNDTLNVYLTAHNAL<br>GSSLHTAGVDLAKSLRIAAKIYSEADEAWRKAIDGLFTGSGGSGGLRGLQGPPGALGPPGSVGSPGSPGPK<br>GQKGDHGDNRAIEEKLANMEAEIRILKSKLQLTNKLHAFSMGGGSGDEDPQIAAHVVSEANSNAASVLQ<br>WAKKGYYTMKSNLVMLENGKQLTVKREGLYYVYTQVTFCSNREPSSQRPFIVGLWLKPSSGSERILLKA<br>ANTHSSSQLCEQQSVHLGGVFELQAGASVFVNVTEASQVIHRVGFSSFGLLKL
LV::S40-EsxH-EsxA-PE19-EspC<br>1R?<br><br>LV::S40-HAPEHR	1004	MLPFLSMLVLLVQPLGNLGAEMKSLSQRSVPNTCTLVMCSPTENGLPGRDGRDGREGPRGEKGDPGLPG<br>PMGLSGLQGPTGPVGPKGENGSAGEPGPKGERGLSGGSGSQIMYNYPAMLGHAGDMAGYAGTLQSLGA<br>EIAVEQAALQSAWQGDTGITYQAWQAQWNQAMEDLVRAYHAMSSTHEANTMAMMARDTAEAAKWG<br>GGSGGTEQQWNFAGIEAAASAIQGNVTSIHSLLDEGKQSLTKLAAAWGGSGSEAYQGVQQKWDATATE<br>LNNALQNLARTISEAGQAMASTEGNVTGMFAGGGSGGSFVTTQPEALAAAAANLQGIGTTMNAQNAAA<br>AAPTTGVVPAAADEVSALTAAQFAAHAQMYQTVSAQAAAIHEMFVNTLVASSGSYAATEAANAAAAG<br>GGSGGTENLTVQPERLGVLASHHDNAAVDASSGVEAAAGLGESVAITHGPYCSQFNDTLNVYLTAHNAL<br>GSSLHTAGVDLAKSLRIAAKIYSEADEAWRKAIDGLFTGSGGSGGTTARDIMNAGVTCVGEHETLTAAAQ<br>YMREHDIGALPICGDDDRLHGMLTDRDIVIKGLAAGLDPNTATAGELARDSIYYVDANASIQEMLNVMEE<br>HQVRRVPVISEHRLVGIVTEADIARHLPEHAIVQFVKAICSPMALASGGGSGSGGLSTISSKADDIDWDAIA<br>QCESGGNWAANTGNGLYGGLQISQATWDSNGGVGSPAAASPQQQIEVADNIMKTQGPGAWPKCSSCSQ<br>GDAPLGSLTHILTFLAAETGGCSGSRDDGGSGGLRGLQGPPGALGPPGSVGSPGSPGPKGQKGDHGDNRA<br>IEEKLANMEAEIRILKSKLQLTNKLHAFSMGGGSGDEDPQIAAHVVSEANSNAASVLQWAKKGYYTMKS<br>NLVMLENGKQLTVKREGLYYVYTQVTFCSNREPSSQRPFIVGLWLKPSSGSERILLKAANTHSSSQLCEQQ<br>SVHLGGVFELQAGASVFVNVTEASQVIHRVGFSSFGLLKL
LV::S40-EsxH-EsxA-PE19-EspC<br>HR-CCL20<br><br>LV::S40 HAPEHR-20	1128	MLPFLSMLVLLVQPLGNLGAEMKSLSQRSVPNTCTLVMCSPTENGLPGRDGRDGREGPRGEKGDPGLPG<br>PMGLSGLQGPTGPVGPKGENGSAGEPGPKGERGLSGGSGSQIMYNYPAMLGHAGDMAGYAGTLQSLGA<br>EIAVEQAALQSAWQGDTGITYQAWQAQWNQAMEDLVRAYHAMSSTHEANTMAMMARDTAEAAKWG<br>GGSGGTEQQWNFAGIEAAASAIQGNVTSIHSLLDEGKQSLTKLAAAWGGSGSEAYQGVQQKWDATATE<br>LNNALQNLARTISEAGQAMASTEGNVTGMFAGGGSGGSFVTTQPEALAAAAANLQGIGTTMNAQNAAA<br>AAPTTGVVPAAADEVSALTAAQFAAHAQMYQTVSAQAAAIHEMFVNTLVASSGSYAATEAANAAAAG<br>GGSGGTENLTVQPERLGVLASHHDNAAVDASSGVEAAAGLGESVAITHGPYCSQFNDTLNVYLTAHNAL<br>GSSLHTAGVDLAKSLRIAAKIYSEADEAWRKAIDGLFTGSGGSGGTTARDIMNAGVTCVGEHETLTAAAQ<br>YMREHDIGALPICGDDDRLHGMLTDRDIVIKGLAAGLDPNTATAGELARDSIYYVDANASIQEMLNVMEE<br>HQVRRVPVISEHRLVGIVTEADIARHLPEHAIVQFVKAICSPMALASGGGSGSGGLSTISSKADDIDWDAIA<br>QCESGGNWAANTGNGLYGGLQISQATWDSNGGVGSPAAASPQQQIEVADNIMKTQGPGAWPKCSSCSQ<br>GDAPLGSLTHILTFLAAETGGCSGSRDDGGSGGGFPGPPGPKGEPGSPAGRGERGFQGSPGKMGPAGSKG<br>EPGGSGSGGASNYDCCLSYIQTPLPSRAIVGFTRQMADEACDINAIIFHTKKRKSVCADPKQNWVKRAVN<br>LLSLRVKKMGSGSGSGGLRGLQGPPGALGPPGSVGSPGSPGPKGQKGDHGDNRAIEEKLANMEAEIRILK<br>SKLQLTNKLHAFSMGGGSGDEDPQIAAHVVSEANSNAASVLQWAKKGYYTMKSNLVMLENGKQLTVK<br>REGLYYVYTQVTFCSNREPSSQRPFIVGLWLKPSSGSERILLKAANTHSSSQLCEQQSVHLGGVFELQAGA<br>SVFVNVTEASQVIHRVGFSSFGLLKL
